# Inhibition of the activity of HIV-1 protease through antibody binding and mutations probed by molecular dynamics simulations

**DOI:** 10.1038/s41598-020-62423-y

**Published:** 2020-03-26

**Authors:** Apoorva Badaya, Yellamraju U. Sasidhar

**Affiliations:** Department of Chemistry, Indian Institute of Technology Bombay, Powai Mumbai, 400076 India

**Keywords:** Computational biophysics, Molecular modelling, Biophysical chemistry, Computational models, Biophysical chemistry

## Abstract

HIV-1 protease is an essential enzyme in the life cycle of the HIV-1 virus. The conformational dynamics of the flap region of the protease is critical for the ligand binding mechanism, as well as for the catalytic activity. The monoclonal antibody F11.2.32 raised against HIV-1 protease inhibits its activity on binding. We have studied the conformational dynamics of protease in its free, inhibitor ritonavir and antibody bound forms using molecular dynamics simulations. We find that upon Ab binding to the epitope region (residues 36–46) of protease, the overall flexibility of the protease is decreased including the flap region and the active site, which is similar to the decrease in flexibility observed by inhibitor binding to the protease. This suggests an allosteric mechanism to inhibit protease activity. Further, the protease mutants G40E and G40R are known to have decreased activity and were also subjected to MD simulations. We find that the loss of flexibility in the mutants is similar to that observed in the protease bound to the Ab/inhibitor. These insights highlight the role played by dynamics in the function of the protease and how control of flexibility through Ab binding and site specific mutations can inhibit protease activity.

## Introduction

The HIV-1 protease belongs to the family of aspartyl protease. It cleaves the newly synthesized polyproteins, which is the vital step to create the mature protein components of an infectious HIV-1 virus^1^. Thus, HIV-1 protease is an essential enzyme in the life-cycle of the HIV-1 virus and a potential target for the structure-based drug design. There are several commercial drugs available in the market against the HIV-1 protease. The success rate of these drugs is low due to the occurrence of drug-resistant mutations in the HIV-1 protease^2^. For example, the thermodynamic integration (TI) and MD simulation studies by Chen and his group suggested that there is a change in the shape and conformation of the binding pocket upon certain drug-resistant mutations and this consequently reduces the binding affinity of inhibitor^3^. Also, conventional method of drug delivery targets active sites and many enzymes with related function may have very similar active sites. This may cause adverse side-effects. Therefore, there is a requirement to generate new generation drugs that can function away from active-site and these are allosteric drugs^4–7^. To identify a new target on HIV-1 protease, there is a need to understand the complete structure and dynamics of HIV-1 protease to inhibits its enzymatic activity. The HIV-1 protease is a homodimeric enzyme with each monomer comprising of 99 amino-acid residues^8^. The active site (residues Asp25, Thr26 and Gly27 from both chains A and B) of the protease is covered by two flaps (residues 43–58) from each chain^9,10^.

F11.2.32 is a monoclonal antibody (mAb) raised against the HIV-1 protease. The peptide P36–46 (^36^MNLPGRWKPKM^46^) corresponding to the epitope/elbow region of the protease binds to the complementarity determining regions (CDRs) of the F11.2.32 Ab. Itadopts a compact β-hairpin like conformation in the peptide P36–46:Ab complex. The epitope/elbow region as part of protease, is highlighted in Fig. S1(iii). This compact β-hairpin conformation does not resemble the more open structure of this segment in free HIV-1 protease. The crystal structure of only P36–45 epitope peptide (^36^MSLPGRWKPK^45^) bound to F11.2.32 Ab is available; the crystal structure of the complex of HIV-1 protease and F11.2.32 Ab is not available^11^. Therefore, only the interactions between the P36–45 epitope peptide and antibody are known. However, the interactions between the protease and antibody are not known. Based on the crystallographic studies of F11.2.32 Ab bound to an epitope peptide (P36–45), it is suggested that F11.2.32 could cause antibody induced structural changes in the protease and this may perhaps inhibit the proteolytic activity of the protease^11^.

Since protease-antibody F11.2.32 interactions are not known as explained earlier, we docked the protease in its crystal structure conformation onto the F11.2.32 Ab and simulated the complex in explicit water^12^. The objective of this study was to understand hydrogen-bonding and salt-bridge interactions between the protease and antibody and we identified the key residues involved in these interactions. We also found that CDRs of the antibody are highly flexible and they adjust themselves to accommodate different conformations of the same epitope sequence (P36–45) in peptide and protein antigens. Thus, our study explained the cross-reactivity of P36–45 peptide and protease with antibody^12^.

There is a large diversity in the flap conformations in the unbound state, fluctuating between the closed, semi-open, and wide-open conformations^13–17^. In the closed/semi-open conformation, the catalytic site is covered by two flaps, and thus restricts the entry of most of the ligands. The semi-open conformation is thought to be the thermodynamically favored state in the ligand-free HIV-1 protease^13,14,18^. The flexibility of the flap is required to facilitate the substrate access to and product release from the active site of the enzyme by an open and close mechanism^10^. The binding of protease substrate to the active site could, in principle, be controlled by restriction in the flap movement and thereby inhibit the activity of the HIV-1 protease^13,19–21^. In this study, the flexibility of the flap and other functionally important regions of the HIV-1 protease are examined upon antibody binding by molecular dynamics simulations to understand the role of dynamics in inhibiting the enzyme activity. We compared the positional fluctuations of free and Ab-bound forms of protease. We also considered the inhibitor (ritonavir) bound form of the HIV-1 protease as a control system where protease activity is inhibited. Our studies suggest that upon antibody and inhibitor binding, the overall dynamics of the protease are quenched, including flap and active site regions. The open and close mechanism of the flap is restricted, which in turn may affect the substrate binding to the active site. Such modulations may affect the functional activity of the HIV-1 protease.

G40E and G40R are among several single missense mutations in the elbow region of both the monomers of the HIV-1 protease, which results in the production of non-infectious virus particles due to decrease in the protease activity upon substitution^22^. We, therefore considered the mutants of HIV-1 protease (G40E and G40R) based on these experimental mutational data and subjected them to MD simulations to examine the effect of mutations on the flexibility of the protease. We find from our simulation results that the mutant proteases too show quenching in their positional fluctuations, which is similar to the quenching observed in Ab-bound and inhibitor-bound proteases. The opening of the flap region is restricted, and overall the residues experienced loss of flexibility. Thus, the single mutations (G40E and G40R) in both the monomers cause the protease dimer to be relatively rigidified. Through hydrogen-bonding and salt-bridges analysis, we tried to identify those interactions, which are responsible for rigidifying the Ab-bound, RIT-bound and mutant proteases.

We further studied the correlation of motions in the protease to understand the protein dynamics and conformational changes in WT-free, Ab-bound, RIT-bound and mutant proteases. It is evident from the correlation map that in WT-free protease the flaps and elbow motions are highly correlated. Many experimental studies also point to the correlation between the flap opening and the downward movement of the elbow in the protease^4,6,23,24^. Also, it is evident from the previous MD simulation studies that restricting the movement of the elbow region limits the conformational dynamics of the flaps^24–28^. Moreover, the elbow region is less susceptible to the mutations^7^. These observations along with our Ab-bound and mutant protease study, reinforce the idea that the site P36–46 (elbow region) in HIV-1 protease can be a promising target for allosteric inhibition. Thus, this study points to a plausible method for allosteric drug control.

## Results

### Restricted opening of flap upon antibody/inhibitor binding or upon mutation

**Figure 1:**
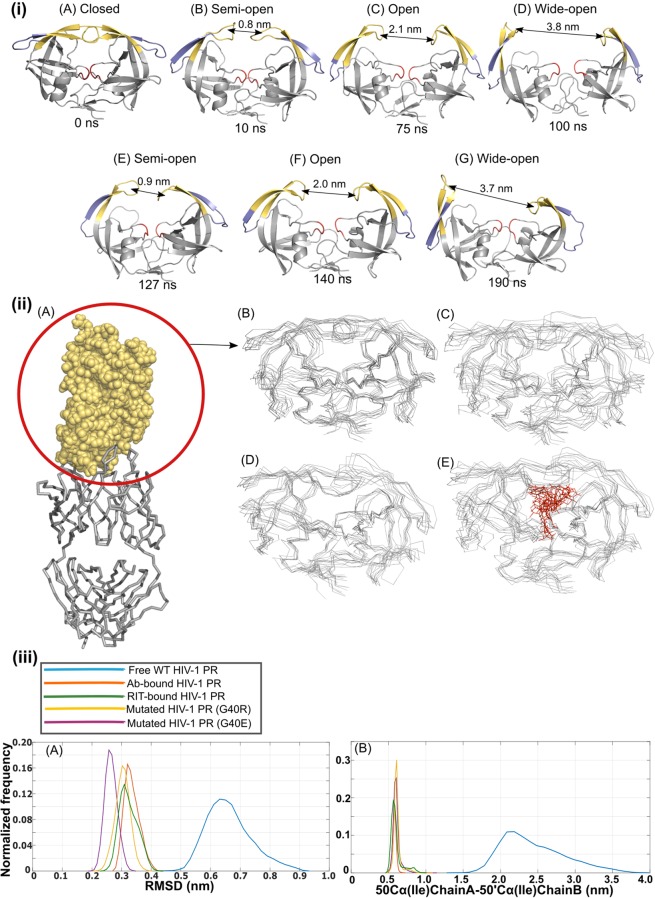
(**i**) The snapshots of the conformation of the HIV-1 protease from the WT-free protease simulation displaying a diversity of flap conformations: **(A)** Closed, **(B**,**E)** Semi-open, **(C**,**F)** Open, **(D**,**G)** Wide-open. **(ii) (A)** The representative conformation of the highly stable complex of the HIV-1 protease (shown in spheres representation) bound to F11.2.32 Ab (shown in trace representation) in the Protease:Ab simulation.**(B)** The superposition of the various conformations sampled by the protease at various time instants (0 ns, 20 ns, 30 ns, 40 ns, 50 ns, 60 ns, 70 ns) in the Protease:Ab complex simulation. **(C,D**,**E)** The superposition of the various conformations sampled by the protease at various time instants (0 ns, 50 ns, 100 ns, 125 ns, 150 ns, 175 ns, 200 ns) in G40E, G40R and RIT-bound (RIT is shown in red in line representation) protease simulations. **(iii)** Normalized frequency distribution plots for, **(A)** backbone RMSD of the HIV-1 protease with respect to its initial conformation, and **(B)** the Cα-Cα distance between the Ile-50 residues present at the tip of the flaps of the protease monomers (50Cα(Ile)chainA-50′Cα(Ile)chainB), for the equilibrated region of the trajectories in the WT-free, mutants, Protease:RIT and Protease:Ab complex simulations.

In our simulation results of free-WT protease for 200 ns, flap region fluctuates between closed (Fig. 1(i)(A)), semi-open (Fig. 1(i)(B),(i)(E)), open (Fig. 1(i)(C),(i)(F)), and wide-open **(**Fig. 1(i)(D),(i)(G)) conformations as shown in Fig. 1(i). We calculated and compared the Cα-Cα distance (flap tip distance) between the Ile-50 residues present at the tip of the flaps of the HIV-1 protease monomers (50Cα(Ile)chainA-50′Cα(Ile)chainB), in the WT-free, Ab-bound, RIT-bound and mutant (G40R and G40E) protease simulations (Fig. 1(iii)(B)). The flap tip distance is assumed to be a reasonable metric to estimate the extent of the flap opening. The flap-tip distance ranging from ~2.0 nm to ~3.0 nm may be considered as an open conformation (Fig. 1(i)(C),(i)(F)). The protease is considered in closed conformation when the flap tip distance is around 0.59 nm^29^ as observed in the crystal structure (see also Fig. 1(i)(A)). The wider opening of the flap with the Cα-Cα flap tip distance > 3.0 nm is also observed in the simulation as shown in Fig. 1(i)(D),(i)(G). However, the populations of wide-open, closed and semi-open conformations sampled in our simulation are rather small.

Upon binding to the Ab or the inhibitor ritonavir (RIT) or upon mutation, we find that the open/close mechanism of the flap of the HIV-1 protease is affected. In Ab-bound, RIT-bound and mutant proteases, the mean flap tip distance value is of about 0.6 ± 0.1 nm, corresponding to closed conformation. Similarly, MD simulation studies by Chen and his group also suggested that most of the time flap tip distance fluctuates between 0.6 nm to 0.8 nm upon binding to the drug Darunavir (DRV) and Amprenavir (APV)^30^. The complex of HIV-1 protease and F11.2.32 antibody is highly stable in the simulation and a representative conformation from the simulation is shown in Fig. 1(ii)(A). Figure 1(ii)(B) displays the superposition of various conformations sampled by the HIV-1 protease at various time instants in the Protease:Ab simulation. It shows that the structure of the protease does not vary much when it is bound to the antibody F11.2.32 and flaps remain in the closed conformation. Similarly, in RIT-bound and mutant proteases (G40E and G40R) also, the structure of protease is stable and the flaps remain in the closed conformation throughout the simulation, as shown in Fig. 1(ii)C,(ii)D,(ii)E.

We also calculated the backbone RMSD of the HIV-1 protease with respect to its initial conformation (Fig. 1(iii)A**)**. The mean RMSD values of the WT-free protease, Ab-bound protease, RIT-bound protease, G40R and G40E mutants of HIV-1 proteases are (0.66 ± 0.07 nm), (0.33 ± 0.02 nm), (0.32 ± 0.03 nm), (0.30 ± 0.02 nm) and (0.26 ± 0.02 nm) respectively. We find that the mean RMSD value is significantly lower for Ab-bound, RIT-bound and mutant proteases as compared to WT-free protease (Fig. 1(iii)A).

The decrease in the values of the RMSD and flap tip distance of the Ab-bound, RIT-bound and mutant proteases on comparing with the WT-free proteases, have further suggested that the flexibility of the protease is reduced in these cases. In other words, the protease has become rigid on Ab or RIT binding. Also, introducing a single mutation at the elbow region affected its flexibility. To further investigate this, we have performed the root mean square fluctuations (RMSF) analysis over the Cα coordinates from the trajectories.

### The quenching of the fluctuations of HIV-1 protease upon Ab/inhibitor binding or upon mutation

**Figure 2:**
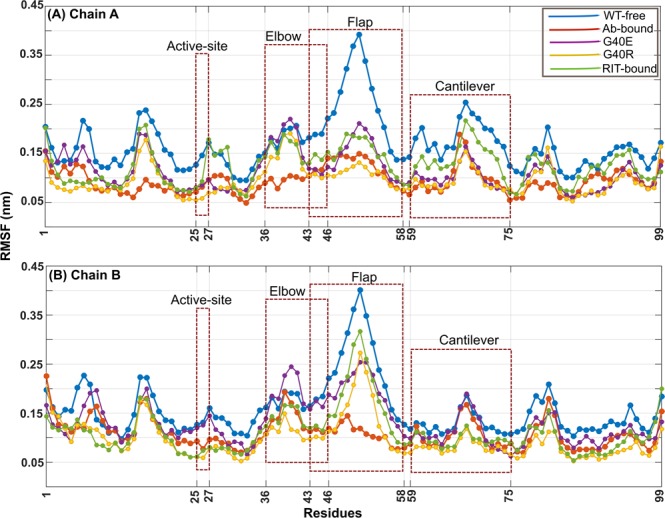
The RMSF for the Cα atoms of the both the chains (**A**,**B**) of HIV-1 protease in the WT-free protease (blue), Protease: Ab complex (orange), Protease:RIT complex(green), G40R (yellow) and G40E (purple) mutant protease simulations.

The root means square fluctuations (RMSF) for the Cα atoms of both the chains of WT-free, RIT-bound, Ab-bound and mutated proteases (G40E and G40R) are computed with respect to the representative structure from the simulation and are compared as shown in Fig. 2. Based on RMSF values, we observed that the overall fluctuations in both the chains of Ab-bound protease are quenched including the functionally important regions such as: (1) Elbow (residues 36–46), (2) Catalytic site (residues 25–27), (3) Cantilever (residues 59–75), (4) Dimer interface (residues 4 to 10 and 90 to 99), and (5) Flaps (residues 43–58). These regions are highlighted in Fig. S1(iii). In mutants and RIT-bound protease also, overall the fluctuations are reduced except the elbow region where change in fluctuation is not that significant. For all these protease systems, the changes in fluctuations are more significant in chain A as compared to chain B. Padariya and Kalathiya also observed in their molecular dynamics simulation study of COM5 ligand bound HIV-1 protease that chain A of protease is more dominant in terms of hydrogen-bonding interactions than chain B, which is also reflected in their RMSF plot^31^. F11.2.32 binds to the elbow region of chain A of HIV-1 protease. Thus fluctuations in the elbow region of chain A of Ab-bound protease are reduced more significantly as compared to the mutants G40E and G40R (Fig. 2(A)). In all the cases, the fluctuations in active-site are considerably reduced (except in chain B of G40E). Along with the RMSF values, the hydrogen-bonding and salt-bridges network also change in the functionally important regions of Ab/RIT bound and mutant proteases, which is discussed in the next section.

### Hydrogen-bonding and salt-bridges network in protease

**Figure 3:**
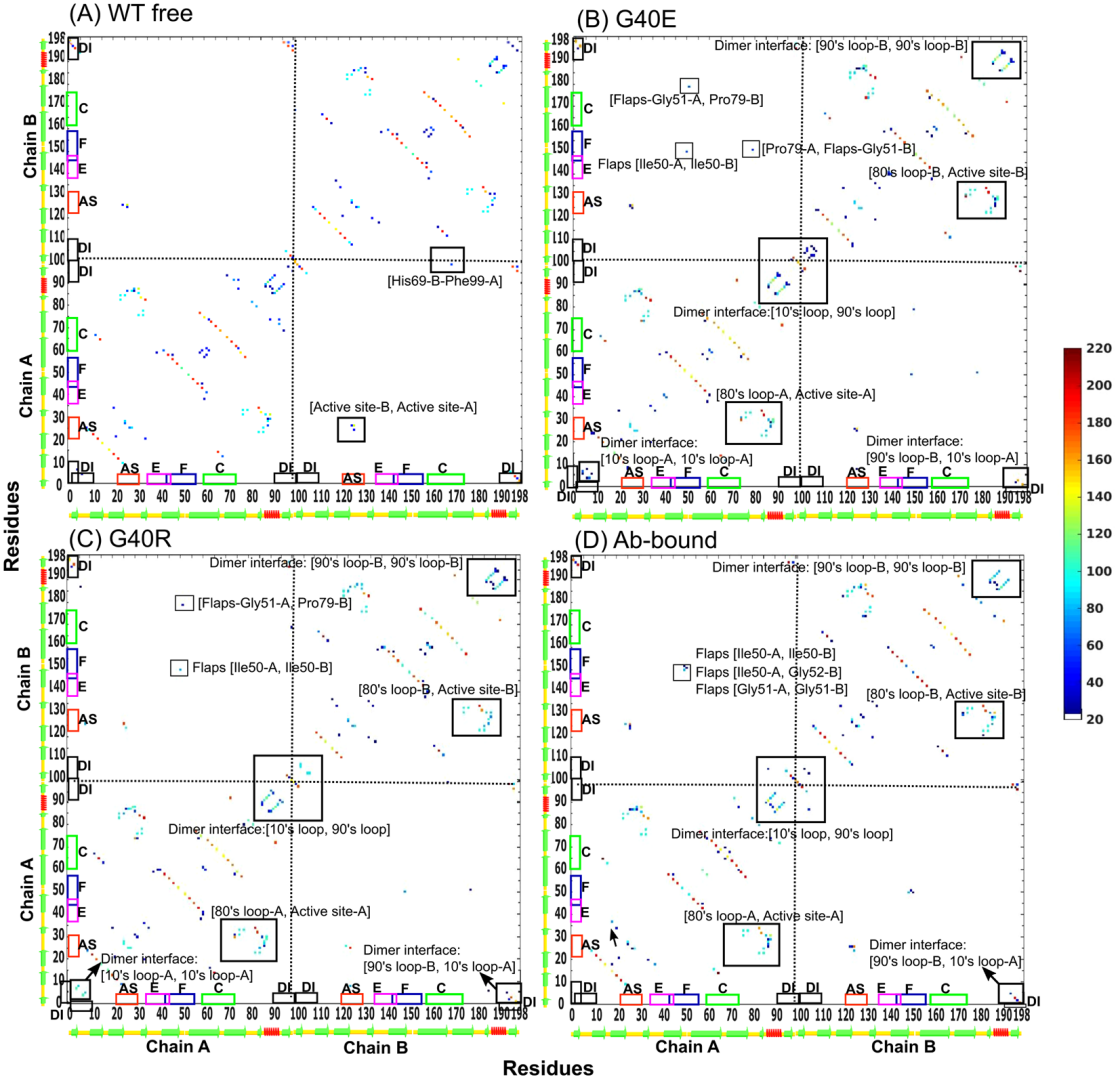
Hydrogen-bonding map computed from the equilibrated region of the trajectories of (**A**) WT-free, (**B**) G40E, (**C**) G40R and (**D**) Ab-bound proteases. Only those hydrogen-bond pairs are shown, which are having a score more than 20 (refer to Methods). The functionally important regions of protease such as dimer interface (DI), active site (AS), elbow (**E**), flaps (**F**) and cantilever (**C**) are marked on axes of the maps. Note that the hydrogen-bonding map for RIT-bound protease is shown in supporting information Fig. S4B.

**Figure 4:**
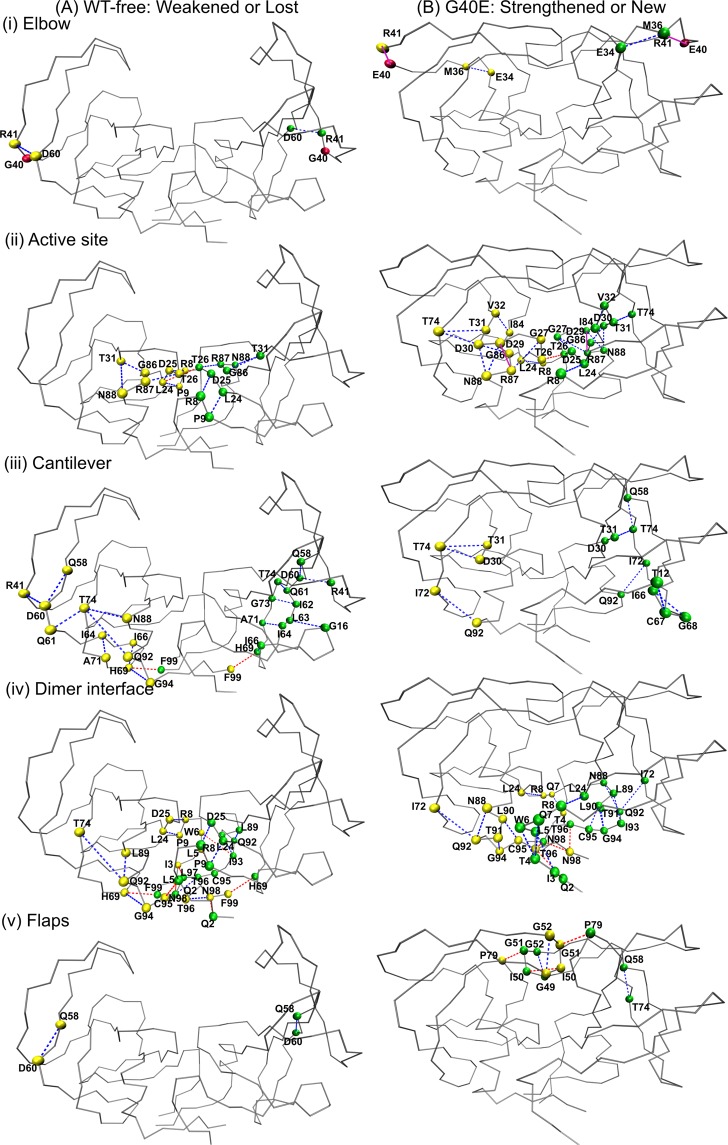
The representative structure of the (**A**) WT-free and (**B**) G40E proteases are shown in trace representation, highlighting and comparing the hydrogen-bonds and salt-bridges that are *weakened or lost* and *strengthened or new* (as explained in Methods) for the functionally important regions individually (**i**) Elbow, (**ii**), Active site (**iii**) Cantilever, (**iv**) Dimer interface and (**v**) Flaps. The Cα atoms of the residues forming the hydrogen-bond are shown in spheres. The hydrogen-bonds within the monomers are shown as blue dashed line, inter-chain hydrogen-bonds are shown in red dashed-line and the salt-bridges are shown in pink solid line.

We have examined the intra protein hydrogen-bonding and salt-bridge network in WT-free, Ab-bound, RIT-bound and mutant proteases (G40E and G40R) simulations to understand the changes in flexibility of protease upon mutation or RIT/Ab binding. The average number of hydrogen-bonds obtained from the distribution of hydrogen-bonds from the mutants (G40R = 134 ± 6, G40E = 139 ± 7), and Ab-bound (132 ± 5) is larger than that of WT-free protease (128 ± 6), even though the width of the distributions for both WT and mutants are similar (the standard deviations obtained from the variances are 6, 7 and 5). So, the increased hydrogen-bonding interactions could underlie the rigidification observed in the mutants and Ab-bound proteases, which corresponds to a decrease in RMSF values, as shown in Fig. 2. Interactions that stabilize RIT-protease complex are described later in this section. The hydrogen-bonding map shown in Fig. 3 displays the sampling of hydrogen-bonds present in the WT-free, Ab-bound and mutated proteases and Fig. S4(B) displays the sampling of hydrogen-bonds present in the RIT-bound protease (refer to Methods). The structural regions like flaps, active site and nearby active-site residues (20–30 s loops), 80 s loop and dimer-interface (10 s and 90 s loop) are labeled in Figs. 3 and S4B to facilitate comparison. As mentioned in Methods, the subtraction of the WT hydrogen-bonding map (matrix) from the mutant/RIT/Ab-bound hydrogen-bonding map (matrix) gives information about the interactions that are *strengthened or new* or *weakened or lost* by mutant/Ab-bound protease with respect to WT-free protease. The hydrogen-bonding scores thus obtained (magnitudes), for the *strengthened or new* hydrogen-bonding interactions are listed in Tables S3–S6 and for *weakened or lost* hydrogen-bonding interactions are listed in Tables S7–S10 (see also figures Figs. 4, S2–S4A). The changes in the hydrogen-bonding networks and salt-bridges in the functionally important regions of HIV-1 protease are discussed in detail as follows.

### Elbow (residues 36–46)

The quenching in the fluctuations of HIV-1 PR is observed, upon binding of the elbow region of the protease to the F11.2.32 Ab (Fig. 2). Similarly, a single mutation in the elbow region of each monomer of the HIV-1 PR has decreased its fluctuations and made the structure rigid (Fig. 2). Mutations at position 40 have resulted in the formation of the salt-bridges between the residues Glu40 and Arg41 in G40E; Arg40 and Asp60 in G40R in both the monomers of the protease as shown in Figs. 4B(i) and S2B(i) (salt-bridge percentages are listed in Table S2). In the case of bound protease, G40 of protease chain A (bound to Ab) forms strong mainchain-mainchain hydrogen-bonding interaction with Arg-31 of the heavy chain of Ab for more than 95% of the time^12^. Due to this strong mainchain-mainchain hydrogen-bonding interaction, the fluctuations in the elbow region of the chain A of Ab-bound protease are presumably reduced more as compared to mutant and WT-free protease (Fig. 2). We find that, in the case of WT-free protease, G40 does not form any interaction with the remaining part of the protease. Thus, it may be surmised that the new salt-bridges at the mutant site (Table S2) and the interactions the elbow region of the Ab-bound protease forms with the Ab, may have resulted in a significant rearrangement of the interactions within the protease.

### Active site (residues 25–27)

The number of salt-bridges and hydrogen-bonding interactions the active site residues (residues 25–27) and the residues near to the active site (Leu24, Asp29, Asp30, Thr31, Val32) are forming with rest of the protease or within themselves are increased in both mutants and Ab/RIT bound proteases (Figs. 4B(ii), S2B(ii), S3B(ii)), S4A(b)(i) and Tables S2–S6**)**. For example, the propensity of the salt-bridges between the residues Asp29 and Arg87 is increased significantly in both the chains of the mutants and Ab/RIT bound protease (Table S2). Many *new and strengthened* hydrogen-bonds are observed in mutants and Ab/RIT bound protease. For example, the following hydrogen bonding pairs are observed between nearby active-site residues (Asp30 and Thr31) and Thr74 of cantilever and Asn88 of 80 s loop for most of the frames: Asp30(A)-Thr74(A), Asp30(B)-Thr74(B), Asp30(A)-Asn88(A), Asp30(B)-Asn88(B), Thr31(A)-Thr74(A), Thr31(B)-Thr74(B) in G40E; Asp30(A)-Thr74(A), Asp30(A)-Asn88(A), Thr31(A)-Thr74(A), Thr31(B)-Asn88(B) in G40R; Asp30(B)-Thr74(B), Thr31(B)-Thr74(B), Thr31(B)-Asn88(B) in Ab-bound; Asp30(A)-Thr74(A), Thr31(A)-Thr74(A), Asp30(B)-Thr74(B), Thr31(B)-Asn88(B) in RIT-bound (Figs. 4B(ii), S2B(ii), S3B(ii)), S4A(b)(i)). Other *new and strengthened* hydrogen-bonding interactions for mutants and Ab/RIT bound proteases along with score are listed in Tables S3–S6. The increase in hydrogen-bonding interactions and salt-bridges involving active site residues can contribute to decreased flexibility; they are no longer as flexible as they were in WT-free protease. This is reflected in their RMSF values as shown in Fig. 2.

Based on the crystal structure of protease bound to inhibitor (PDB id: 1HXW), the residues of the protease, which are involved in forming the hydrogen-bonding interactions with the inhibitor/substrate, are Asp25, Gly27 and Asp30 of chain A and Asp25, Gly27, Asp29 and Gly48 of chain B. We have examined the hydrogen-bonding interactions and hydrophobic contacts, RIT forming with HIV-1 protease in Protease:RIT complex simulation. We find that RIT forms hydrogen-bonding interactions with Gly48 of chain A and Asp25 of chain B of HIV-1 protease (Fig. S4C). These two residues belong to flap and active site regions respectively. The residues which are involved in forming hydrophobic contacts belong to active-site, flaps and 80 s loop such as residues Ala28, Val32, Ile47, Ile50, Ile54, Ile84, Pro81, Val82 of both chain A and chain B **(**Fig. S4D**)**. These hydrogen-bonding interactions and hydrophobic contacts are together responsible for holding the RIT to the active site.

In mutants and Ab-bound protease, instead of substrate analogue, active site and nearby-active-site residues form hydrogen-bonding interactions and salt-bridges among themselves and with rest of the protease, which involves many interactions with the 80 s loop (Figs. 4B(ii), S2B(ii), S3B(ii)). According to the previous studies^14,32–34^, the 80 s loop forms hydrophobic contacts with flaps, which causes the flap to remain closed. In our study also, we find an increase in hydrophobic contacts between 80 s loop and flaps in Ab-bound and mutant proteases (Table S1). Thus, an increase in hydrophobic contacts between flap residues (residues 43–58) and 80 s loop has an interesting correlation with an increase in hydrogen-bonding interactions between active-site/nearby-active-site residues (residues 24–32) with 80 s loop. They mutually strengthen each other’s interaction resulting in the rigidification of the region, which is discussed in detail in discussion.

### Cantilever (residues 59–75)

As suggested by many reports^24,35^, in WT-free protease, flap opening is accompanied by the concerted downward motion of the elbow (residues 36–46), fulcrum (residues 10–23) and cantilever (residues 59–75). In our WT-free protease simulation also, we observed a similar occurrence. But, in mutants and Ab/RIT bound protease, along with flaps, the downward motions of elbow and cantilever are also restricted. The residue His69 is present at the tip of cantilever and Phe99 is the terminal residue of protease; the distance between these two residue pairs His69(A)-Phe99(B) and His69(B)-Phe99(A) is assumed to be a reasonable metric to determine the downward motion of the cantilever as shown in Fig. S5. In WT-free protease simulation, where the protease is mostly in open conformation, the mean distance between these residue pairs His69(A)-Phe99(B) and His69(B)-Phe99(A) are about 0.8 ± 0.1 nm. In G40E mutant and Ab bound protease, the mean distances between these two residue pairs His69(A)-Phe99(B) and His69(B)-Phe99(A) are about 1.2 ± 0.1 nm. In G40R and RIT-bound protease the mean distance between residue pair His69(A)-Phe99(B) is 1.2 ± 0.1 nm and His69(B)-Phe99(A) is about 0.8 ± 0.1 nm. This suggests that when the flaps are closed in mutants and Ab-bound proteases, the downward motion of cantilever is also restricted or decreased.

The following pairs are forming hydrogen-bonding interactions in WT-free protease in which the residues involved belong to the cantilever (Thr74, His69, Gln61) and dimer-interface (Gln92, Gly94, Phe99): Thr74(A)-Gln61(A), Thr74(A)-Gln92(A), His69(A)-Gly94(A), His69(A)-Phe99(B). These interactions are *weakened or lost* in the mutant and Ab/RIT bound protease (Figs. 4A(iii), S2A(iii), S3A(iii) and S4A(a)(ii)). In mutants and Ab/RIT bound protease, the residue Thr74 of the cantilever forms hydrogen-bonding interactions with the core (nearby active-site residues Thr31, Asp30) of the protease. These interactions are *strengthened or new* with respect to WT-free protease **(**Figs. 4B(iii), S2B(iii), S3B(iii) and S4A(b)(ii)). The increase in the interaction of the residues of the cantilever with the core of a protease in mutant and Ab/RIT bound protease, could be a possible reason for rigidifying the active site, which is part of the core of the protease.

### The dimer interface residues

In this section, we will talk about chain A-A, chain B-B and chain A-B interactions close to the dimer interface. The mutation at residue 40 in both the chains of HIV-1 protease has caused many *new and strengthened* interactions involving the protein dimer interface residues (residues 4 to 10 and 90 to 99 in both chains A and B). The examples of some new *and strengthened* interactions formed by the mutants with respect to WT-free protease are given as follows. The hydrogen-bonding interactions formed within the dimer-interface residues are: Cys95-Leu90, Gly94-Thr91, Gln7-Thr4 in chain A and Cys95-Leu90, Gly94-Leu90, Gly94-Thr91, Gln7-Thr4 in chain B of both the mutants; the hydrogen-bonding interactions between dimer-interface residues and residues belonging to 80 s loop/cantilever are: Gln92-Asn88, Gln92-Ile72 in chain A and Ile93-Leu89 in chain B of both the mutants; the interchain interactions formed by the dimer-interface residues are Thr96(A)-Gln2(B), Asn98(A)-Asn98(B), Thr96(A)-Asn98(B) in G40E and Gln2(A)-Thr96(B) Asn98(A)-Asn98(B) and Asn98(A)-Thr96(B) in G40R (Figs. 4B(iv) and S2B(iv)). They are listed in Tables S3 and S4 and are shown in the hydrogen-bonding map in Fig. 3. These *new and strengthened* hydrogen-bonds in the dimer interface upon mutation at elbow site, suggests a direct correlation of the 90 s and 10 s loop with the elbow, which is also reflected in cross-correlation plots shown in Fig. S6, and discussed in detail in Discussion.

### Flaps (residues 43–58)

The flaps display an open/close mechanism in WT-free protease (Fig. 1(i)). This open/close mechanism is restricted in mutants and Ab/RIT bound proteases and the flaps remain closed throughout the simulation (Fig. 1(ii)). *New* hydrogen-bonds are observed in flaps of Ab/RIT bound protease and mutant proteases (G40E and G40R) as shown in Figs. 4B(v), S2B(v), S3B(v) and S4A(b)(iv). In both the mutants, some of the new hydrogen-bonding interactions the flaps form within themselves are, Gly49(A)-Gly52(A) and Ile50(A)-Ile50(B). In mutants, some new hydrogen-bonds are also formed between flaps and remaining parts of the protease such as Gln58(A)-Thr74(A), Gly51(A)-Pro79(B) in G40R and Gln58(B)-Thr74(B), Pro79(A)-Gly51(B) and Gly51(A)-Pro79(B) in G40E. Similarly, the *new* interchain hydrogen-bonds formed between flaps by Ab-bound protease are Ile50(A)-Ile50(B), Gly51(A)-Gly51(B), Ile50(A)-Gly52(B) and by RIT-bound protease are Ile50(A)-Ile50(B), Gly52(A)-Ile50(B). These interactions help in keeping the flaps in closed conformation. The hydrogen-bonding scores are listed in Tables S3–S6. The increase in hydrogen-bonds in flaps of mutants and Ab/RIT bound protease correlates well with the decrease in the RMSF value in both the chains of protease as shown in Fig. 2.

## Discussion

### The overall structure of the HIV-1 protease is rigidified upon Ab/RIT binding or upon mutation

HIV-1 protease is widely studied by techniques including nuclear magnetic resonance (NMR)^36–40^, X-ray crystallography^41–43^, electron paramagnetic resonance (EPR)^17,20^, and molecular dynamics simulations^23,35,44,45^. NMR experiments suggested large scale flap dynamics of unliganded form of HIV-1 protease. For example, Torchia and coworkers suggested the larger motions of the tip of flaps occur on a microsecond time scale while the rapid local fluctuations occur at nanosecond time scales^38,40,46^. The crystal structures of unbound HIV-1 proteases are displaying diversified conformations in terms of flap conformations ranging from closed to semi-open forms, including a small population of wide-open conformations^42,47,48^. This heterogeneity in data could be due to crystal packing effects^42,47,48^. Also, the sequence variation of various subtypes of HIV-1 protease could be a probable reason behind the variations observed in flap conformational diversity^43,49^.

Simulations studies are mostly carried out at a nanosecond time scale. Early MD studies suggested that the closed conformation of ligand-free HIV-1 protease is sampled predominantly in solution^50^ while the recent studies like all atom or coarse-grained MD simulations observed spontaneous opening and reclosing of the flaps^13–17,44,45^. Simmerling and his group based on their double electron-electron resonance (DEER), electron paramagnetic resonance (EPR) and MD simulations studies, suggested a diverse ensemble of conformations of unbound HIV-1 protease, fluctuating between semiopen, closed and fully open conformations^7,20,35^. In their 30 ns simulation study of HIV-1 protease, they observed large flap openings, with a flap tip distance of around 3.0 nm^35^. Extensive sampling of opening and closing events were also observed by Tozzini and his group in a molecular dynamics simulation study on the time scale of tens of microseconds using a coarse-grained model of HIV-1 protease^44^. They observed 4 different conformations of HIV-1 protease, namely closed, semi-open, open, wide-open with a flap-tip distance of ~0.5 nm, ~0.12 nm, ~2.0 nm and ~3.5 nm respectively^44^. This is similar to what we observed in our WT-free protease simulation study. In our simulation of WT-free protease, we observed open conformation as the predominant conformation with flap-tip distance ranging from ~2.0 nm to ~3.0 nm most of the time (Fig. 1(iii)(B)). Semi-open and wide-open conformations are also sampled, but by a relatively small population (Fig. 1(iii)(B)). However in case of Ab-bound, RIT-bound and mutant proteases mean flap tip distance is about 0.6 ± 0.1 nm. This value compares well to 0.6 nm observed for flap-tip distance in the crystal structure of WT-protease (PDB id: 1HXW) in closed conformation. (Fig. 1(iii)B). RIT-bound protease is seen to be behaving like Ab-bound and mutant proteases in terms of fluctuations and dynamics. Moreover, these systems (Ab/RIT bound protease and mutants) have very similar *weakened or lost*/*strengthened or new* hydrogen-bonding interactions and salt-bridges as given in Results. Thus, mutant proteases and Ab-bound protease are emulating the effect of inhibitor binding. Some NMR and MD studies also suggest that, while, free protease displays the large flap dynamics at various time-scales, the flaps become rigidified upon binding of the ligand^35,40,46^.

As it is known, the flexibility of the flaps is required for the proper functioning of the protease since it controls the access of the substrate to the active site by open/close mechanism^19^. Mittal, S. *et al*. tested the role of flexibility in protease activity by constructing two protease variants by introducing a pair of cysteines (G16C/L38C and R14C/E65C) at the interfaces of flexible regions remote from the active site in both the chains of protease^51^. The introduction of disulfide bonds decreases the flexibility and lowers the enzyme activity by 146 fold in G16C/L38C variant. This suggests, the changes in flexibility, affects the activity of the protease.

### Mechanism of Ab-bound, RIT-bound and mutant proteases inhibition

Through internal hydrogen-bonding and salt-bridges network analysis in WT-free, RIT-bound, Ab-bound and mutant proteases (G40E and G40R) simulations, we find a significant rearrangement of hydrogen-bonding interactions and salt-bridges in protease due to mutation and Ab/RIT binding (Figs. 4, S2–S4A). The interactions that are *strengthened or new* or *weakened or lost* by mutant or Ab/RIT bound protease with respect to WT-free protease are observed in the functionally important regions of the protease, which include elbows, active-site, cantilever, flaps and dimer-interface as discussed earlier in Results. In WT-free protease, the cantilever residue Thr74 forms some hydrogen-bonding interactions with rest of the protease (Gln61, Gln92, Asn88), which seem to be holding flap in open conformation. These interactions are *weakened or lost* in mutants and Ab/RIT bound protease as shown in Figs. 4A(iii), S2A(iii), S3A(iii), S4A(a)(ii). The cantilever residue Thr74 forms *strengthened and new* hydrogen-bonding interactions with the nearby active-site residues (Asp 30 and Thr31) in mutants and Ab/RIT bound protease as shown in Figs. 4A(iii), S2A(iii), S3A(iii), S4A(a)(ii). We surmise that the *weakened* and *lost* interactions by residue Thr74 in mutants and Ab/RIT bound protease are causing the flaps to release from open conformation; the *strengthened* and *new* interactions formed by residue Thr74 in mutants and Ab/RIT bound protease could be responsible for holding the flaps in closed conformation.

Further, in accordance with the previous studies^14,32–34^, in our RIT-bound, Ab-bound and mutant protease simulations also, we find an increase in the hydrophobic contacts between the 80 s loop and flap residues; and between the two flaps themselves with respect to WT-free protease **(**Table S1**)**. For example, Ile50 of chain A forms hydrophobic contact with Ile84 of chain B for about 95% of the time in Ab-bound and G40E mutant protease and about 86% of the time in G40R protease. Similarly, Ile54 of chain A forms hydrophobic contact with Ile50 of chain B for about 95% of the time in Ab-bound, G40R and G40E protease simulations and 88% of the tine in RIT-bound protease simulation. These hydrophobic contacts are almost absent with less than 10% sampling in WT-free protease. Other hydrophobic contacts are listed in Table S1, which are formed between 80 s loop and the flaps and between the flaps themselves.

This increase in hydrophobic contacts in 80 s loop and flaps correlates well with the increase in the number of salt-bridges and hydrogen-bonding interactions the active site residues and the residues near to the active site are forming with 80 s loop as mentioned earlier in Results (Figs. 4B(ii), S2B(ii), S3B(ii) and S4A(b)(i)). It may be surmised that an increase in hydrogen-bonding interactions and salt-bridges make the 80 s loop rigid, which therefore, facilitates the hydrophobic contacts between 80 s loop and the flaps and between the flaps themselves, which are absent in WT-free protease. The hydrogen-bonding interactions are also formed between the flaps of both the chains as already mentioned in Results. Thus, this increase in hydrophobic contacts and hydrogen-bonding interactions may encourage the flap to remain closed and thereby inhibiting the activity of protease.

### Ab/RIT bound and mutated proteases reveal reduction/attenuation of correlations in some regions

The opening of the flap (residues 43–58) results in the downward movement of the elbow (residues 36–46), fulcrum (residues 10–23) and cantilever (residues 59–75) regions^24,35^. This suggests the possibility of having a correlation in their motions. The protein dynamics in WT-free, RIT-bound, Ab-bound and mutant proteases were further explored by studying the correlation of motions between the residues of the protease as shown in Fig. S6. The cross-correlation coefficient matrix analysis was performed for the Cα backbone atoms of the WT-free, RIT-bound, Ab-bound and mutant proteases. The information about the correlated motions of Cα atom pairs is provided by the cross-correlation coefficients (see Methods). A point mutation in elbow region causes rearrangement of hydrogen-bonds in active-site, dimer-interface and flaps regions as discussed earlier. This mutation also causes significant changes in the cross-correlation of these regions with elbow region. For example, in WT-free protease, active-site and dimer-interface residues (10 s loop and 90 s loop) have a negative or anti-correlation with residues in elbow region (Fig. S6A). This correlation is reduced in mutants, RIT-bound and Ab-bound proteases (Fig. S6B–D). Similarly, there is a positive cross-correlation between flaps and elbow residues of the same chain and negative cross-correlation between flaps and elbow residues of different chain in WT-free protease. This correlation between flap and elbow of same and different chains in mutant, RIT-bound and Ab-bound proteases is either lost or reduced for some residue pairs and for some residue pairs the correlation are reversed. In the same way, as compared to WT-free protease, the correlation for a few residues between the flaps of two chains are changed from negative to positive in mutant, RIT-bound and Ab-bound proteases and for remaining residues the correlations are lost. These correlations are highlighted in Fig. S6.

The Ab or RIT binding and mutation at elbow region of HIV-1 protease causes the structure to be rigidified (Fig. 1(ii)). At the same time there is a significant reduction in the correlations involving some residue pairs as described above and the Ab-bound, RIT-bound and G40E/G40R mutated proteases adopt the closed inactive conformation. Previous studies^52,53^ also suggested that the correlations are decreased in the protein upon inhibitor binding. For example, the open form of HCV NS3/4 A represents the active state and inhibitor bound HCV NS3/4 A represents closed inactive state. The apo form of HCV NS3/4 A shows higher correlations and there is a rapid reduction in the correlation of motions of inhibitor-bound HCV NS3/4 A^52^. Similarly in Zika NS3 helicase, the conformational shifts are observed after ligand binding and globally more correlated motion is observed in the case of the free protein^53^.

### Elbow region (P36–46): Probable allosteric target in the HIV-1 protease

The conventional method of drug delivery may result in adverse side effects because many enzymes with related functions may have very similar active sites^7^. The similar conformation of the active site may lead to a lower specificity of a drug for the desired protein. Hence, the method of targeting the active sites by the drugs can often be harmful. Changeux introduced the concept of allosterism in 1965^5^. According to this model, effector ligands bind to the sites, which are located away from the active sites. Such sites are termed as allosteric sites. The ligands, upon binding to the allosteric site, can modulate the activity of the target protein. It is expected that allosteric site based drug discovery may lead to more effective therapeutic agents with fewer side effects^7^.

The use of allosteric modulation to inhibit the enzyme activity has increased dramatically in the past decade^5,25^. The allosteric ligands have been developed for numerous therapeutic targets, including ion channels, caspases, kinases, phospholipases, and GPCRs^5,25^. The allosteric site can be a useful target, firstly, if it is unique in the protein of interest, and secondly, if it can alter the functional properties of the protein when a ligand binds to this site^4^.

**Figure 5:**
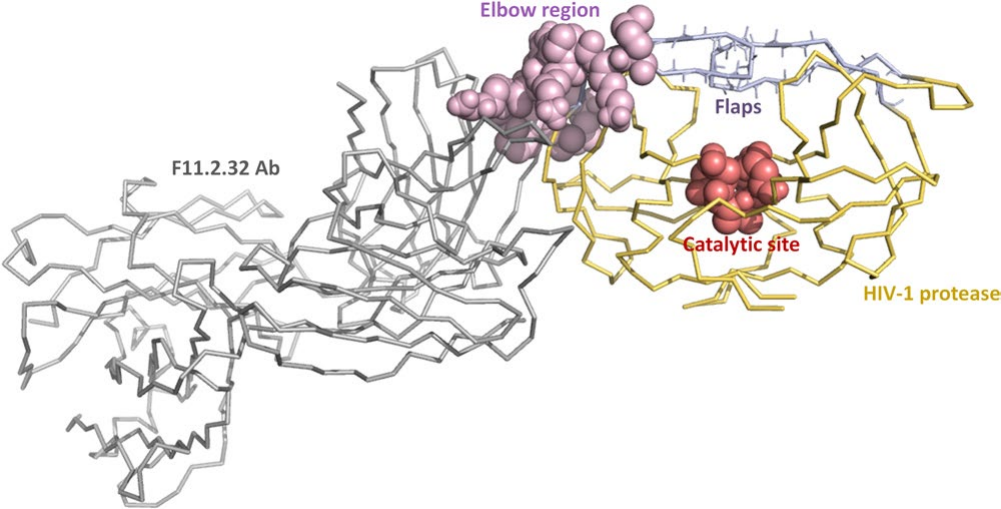
The representative conformation of docked complex of HIV-1 protease and F11.2.32 Ab obtained from Protease:Ab simulation. F11.2.32 Ab (grey) binds to the elbow region (pink) of the HIV-1 protease and affects the global conformation and dynamics of HIV-1 protease (yellow) including the flaps (light blue) and the active/catalytic site (red). The elbow and catalytic region are shown as spheres, and the remaining part of the complex is shown in trace representation.

In case of HIV-1 protease, the elbow region (residues 36–46) could be a probable allosteric site for drug binding (Fig. 5)^23–28^. The correlation between the elbow and the flap regions of the HIV-1 protease are cited in many reports^23–28^. It is seen from the literature that restricting the movement of the elbow region limits the conformational dynamics of the flaps^24,25^. The CDRs of the Ab, bind to the epitope region P36–46 (elbow region) of the protease (Fig. 1(ii)). Upon binding, the quenching of the fluctuations is observed in all the residues of the HIV-1 protease (Fig. 2). This is an evidence of allosteric control, where the Ab contacts to the elbow region of the HIV-1 protease and causes the flaps to lock down their conformational changes and decreases the overall flexibility of the HIV-1 protease including active site, cantilever, elbow and the flap regions. In G40E and G40R mutants also, the single mutation (G40E/G40R) in elbow region of both the monomers causes the protease dimer to rigidified and the opening of the flap region is restricted. Thus, the elbow region can be considered as an allosteric site for drug binding, where an antibody or antibody-mimetic can bind, which can modulate the functional activity of HIV-1 protease. Such novel drug discovery methods may increase the chances of obtaining more effective drugs with fewer side effects.

## Methods

### System for simulation

From our previous work^12^, we have used the data of the molecular dynamic simulations of the following systems in an explicit SPC^54,55^ water at 300 K:

**Table:** 

• WT-free (set 1)	Free Wild-type HIV-1 protease dimer (200 ns)
• Protease:Ab complex	Docked HIV-1 protease-Ab complex (75 ns)

We have additionally performed the molecular dynamic simulations on the following systems in explicit SPC water at 300 K.

**Table:** 

• WT-free (set 2)	Free Wild-type HIV-1 protease dimer (200 ns)
• WT-free (set 3)	Free Wild-type HIV-1 protease dimer (200 ns)
• G40E HIV-1 protease (mutant)	Dimer of free HIV-1 protease with mutation of the residue G40 with glutamate (E) in both the monomers (200 ns)
• G40R HIV-1 protease (mutant)	Dimer of free HIV-1 protease with mutation of the residue G40 with arginine (R) in both the monomers (200 ns)
• Protease:RIT complex	Wild-type HIV-1 protease bound to Ritonavir inhibitor (200 ns)

The simulations were performed on dual Xeon quad-core processor based machines with CentOS 7.0 operating system (http://www.centos.org/) installed on them using the GROMACS 5.0.2 software package and GROMOS96 54a7^56^ force field. The initial structure is crystallographic structure of the wild-type HIV-1 protease (PDB ID: 1HXW)^29^ obtained from the Protein Data Bank (PDB)^57^ (www.rcsb.org). The ligand ritonavir (RIT) was removed from crystal structure. We performed 3 sets (S1, S2 and S3) of simulation of WT-free HIV-1 protease for 200 ns each, where the set S1 data was from a previous study of ours^12^. The ‘*gen-seed’* which is used to obtain different random velocities was kept different for each set. Thus each set has different initial velocities. Rest of the parameters were kept same. The equilibrated portion of the trajectories from sets 1 to 3 (Fig. S1(i)C) were concatenated and used for analysis. We performed another simulation of HIV-1 protease bound to ritonavir (RIT) inhibitor as a control system where protease activity is inhibited. The initial structure of WT HIV-1 protease bound to RIT obtained from PDB (PDB ID: IHXW)^29^. The PRODRG server^58^ was used to generate topology parameters for RIT for GROMOS96 54a7 force field. Based on available information in literature^59,60^, we have kept the active site residue Asp 25 from both the chains of HIV-1 protease in ionized state. To build the initial structure of the mutants (G40E and G40R) of HIV-1 protease, the crystallographic structure of the wild-type HIV-1 protease (PDB ID: 1HXW)^29^ obtained from the Protein Data Bank (PDB)^57^ (www.rcsb.org) was taken as a reference. The residue glycine at position 40 in each monomer of HIV-1 protease was mutated to residue glutamate in G40E mutant and residue arginine in G40R mutant by using the mutation tool in the DeepView software^61^.

The simulations of WT-free, Protease:RIT complex and mutants were performed in a cubic box. All the systems were solvated with SPC^54,55^ water molecules and neutralized using counter ions (Cl^−^ and Na^+^). The WT-free and mutant systems were subjected to energy minimization using the steepest descent algorithm for about 400 steps with a force tolerance value of 100 kJ mol^−1^ nm^−1^. The potential energy was lowered within the specified upper limit of 400 steps. To obtain further convergence, the conjugate gradient method was used subsequently for 1000 steps using the force tolerance value mentioned above. The Protease:RIT system was energy minimized both in vacuum and solvent using steepest descent followed by conjugate gradient method using the same parameters described above. The energy minimization was repeated for 3 times for Protease:RIT system. This procedure ensured stability of the complex in simulation. The electrostatic interactions were treated using Particle Mesh Ewald (PME) algorithm^62^ with a coulomb cutoff of 1.1 nm and the van der Waal’s interactions were treated using the Lennard-Jones potential and switching function with a cut-off of 1.1 nm and a switching distance of 0.8 nm. The system was equilibrated for 500 ps using NVT (constant volume and temperature) ensemble by keeping the protein atoms restrained to fixed positions. After equilibration, the final MD was performed with an integration time step of 2 fs and the LINCS algorithm^63^ was used to constrain all the bonds. The positions and the velocities of the atoms were saved every 0.5 ps in a trajectory file. Temperature coupling was used to keep the temperature at 300 K using Berendsen’s thermostat^64^ with a time constant of 0.1 ps. Various simulation parameters like box dimensions, etc., are summarized in Table 1.

**Table 1:** Lists the details about the protein systems used in the current simulation study.

Peptide or protein systems	Temp. (K)	Length of the simulation (ns)	No. of peptide and/or protein residues	No. of SPC water molecules	Total system size in atoms	Box type	Box dimensions (nm)
Mutant HIV-1 protease (G40E)	300	200	198	24685	75973	Cubic	9.24
Mutant HIV-1 protease (G40R)	300	200	198	24681	75976	Cubic	9.24
WT-free (set 2)	300	200	198	24974	76832	Cubic	9.24
WT-free (set 3)	300	200	198	24974	76832	Cubic	9.24
Protease:RIT complex	300	200	198	24933	74804	Cubic	9.24

All the analysis were performed using the tools incorporated in the GROMACS software package. The graphs and figures were made using MATLAB (http://www.mathworks.com) and PyMOL (http://www.pymol.org/) software.

### Method for convergence check

To check the convergence of the trajectory of the G40E, G40R, Protease:RIT complex and 3 sets of WT-free HIV-1 protease, the variation of the sampling frequency of the first two most populated clusters was determined using gromos method, with respect to simulation time with an RMSD cut-off of 0.22 nm, 0.20 nm and 0.27 nm respectively. These cut-off values were determined from pairwise rmsd distributions in the respective simulations. The overlapping periods such as 0–10 ns, 0–20 ns, 0–30 ns and so on up to 0–200 ns were used to determine sampling frequency of clusters. The membership of cluster1 and cluster 2 was calculated, which is the fraction of frames in cluster 1 or cluster 2 with respect to the total number of frames as shown in Fig. S1(i). As can be seen, the frequency of cluster sampling is stabilized from ~100 ns onwards in G40R and WT-free HIV-1 protease simulations (3 sets) and 130 ns onwards in G40E and Protease:RIT complex simulation, indicating equilibration of the MD trajectories. The RMSD values and centre of mass (COM) distance between the monomers of protease for the G40E, G40R and WT-free HIV-1 protease are also stable throughout the simulation (Fig. S1(ii)). The equilibrated regions of MD trajectories of mutants and Protease:RIT were used for analysis and concatenated trajectory of the 3 sets of WT-free protease is used for analysis as already mentioned above. For, Ab-bound protease, the equilibrated region (45–75 ns) of Protease: Ab complex simulation^12^ was used for analysis. We clustered the frames from the equilibrated region of the trajectories and selected central conformation from the most populated cluster as representative conformation.

### Hydrogen-bonding analysis

The equilibrated regions of MD trajectories were used to determine the hydrogen-bonding pairs and salt-bridges present in the WT-free, mutant, RIT-bound and Ab-bound proteases. To calculate hydrogen-bonds, the cut-off used to calculate the donor-acceptor distance is ≤ 0.35 nm and Hydrogen-Donor-Acceptor angle is 30 degrees as per default options of gromacs tool *gmx hbond* (http://manual.gromacs.org/documentation/2018/onlinehelp/gmx-hbond.html). These hydrogen-bond donor-acceptor pairs are identified using a script available at http://www.bevanlab.biochem.vt.edu/Pages/Personal/justin/Scripts. The two-dimensional hydrogen-bonding maps were computed for WT-free, mutants, RIT-bound and Ab-bound proteases, as described below, to display the hydrogen-bonding pairs present in the protease using our own scripts running in MATLAB.

#### Hydrogen-bonding map

The horizontal and vertical axes of the hydrogen-bonding map represents the amino acid residue numbers that are involved in forming the hydrogen-bonding interactions. A point on the hydrogen-bonding map indicates the hydrogen-bonding interactions between the two residues. The total percentages of hydrogen-bonds between the two residues are calculated and added. For example, if N of Ile66 is forming hydrogen-bond with O of His69 91% of the time and O of Ile66 is forming hydrogen-bond with N of His69 78% of the time, the total score for forming the hydrogen-bond between Ile66 and His69 becomes 169. Thus, this hydrogen-bonding score (score more than 20) is plotted onto the hydrogen-bonding map and color-coded from dark-blue (less score) to red (high score).

The hydrogen-bonding maps are compared for WT-free, mutant, RIT-bound and Ab-bound proteases to understand the rearrangement of hydrogen-bonds upon mutation or binding to the Ab/RIT. We frequently refer to the functionally important regions of protease such as: (1) Elbow (residues 36–46), (2) Active/catalytic site (residues 25–27), (3) Cantilever (residues 59–75), (4) Dimer interface (residues 4 to 10 and 90 to 99), and (5) Flaps (residues 43–58). These regions are highlighted in Fig. S1(iii).

To identify salt-bridge pairs in equilibrated portion of trajectory, VMD saltbridges plugin (https://www.ks.uiuc.edu/Research/vmd/plugins/saltbr/) was used and gmx distance tool of GROMACS was used to calculate sampling frequency of identified pairs. A salt-bridge is considered to be present when the distance between N and O atoms of the charged residue side-chains is less than or equal to 0.4 nm^65^. If the cumulative salt-bridge sampling between two residues is more than 20% only, such a salt-bridge is considered for analysis.

### Changes in hydrogen-bonding interactions in the mutant and Ab/RIT bound proteases with respect to WT-free protease

The subtraction of the WT hydrogen-bonding map (matrix) from the mutant/RIT-bound/Ab-bound hydrogen-bonding map (matrix) gives information about the interactions that are *strengthened or new* (positive values of difference score) or *weakened or lost* (negative values of difference score) by mutant/RIT-bound/Ab-bound protease with respect to WT-free protease. The score cut-off used to identify these interactions is 20, as discussed below.

#### Strengthened

The hydrogen-bonding interactions, which are sampled in mutant/RIT-bound/Ab-bound with a score of ≥ 20 than those in the WT-free protease.

#### New

The hydrogen-bonding interactions, which are not present in WT-free protease but newly appeared in mutant/RIT-bound/Ab-bound with a hydrogen-bonding score of ≥ 20.

#### Weakened

The hydrogen-bonding interactions, which are sampled in mutant/RIT-bound/Ab-bound with a score of ≤ 20 than those in the WT-free protease.

#### Lost

The hydrogen-bonding interactions that are present in WT-free protease are considered lost in mutant/Ab/RIT bound if their hydrogen-bonding score is ≤ 20 in the mutant or Ab/RIT bound protease or if this hydrogen-bond is absent in the mutant or Ab/RIT bound protease.

There are hardly any salt-bridges sampled in WT-free protease and these are observed mainly in mutant or bound proteases. These are labeled as *strengthened or new* in all the figures for the sake of uniform labeling of hydrogen-bonding and salt-bridge interactions. A significant rearrangement of hydrogen-bonding interactions are observed upon mutation or Ab/RIT binding to HIV-1 protease, and hence, is responsible for the rigidity of the protease. Thus, overall we are focusing only on *strengthened or new* or *weakened or lost* hydrogen-bonding interactions and salt-bridges.

In the functionally important regions of protease, these hydrogen-bonding interactions and salt-bridges identified as described above, are depicted onto the representative structures, obtained from the WT-free, mutant, RIT-bound and Ab-bound proteases simulations. *Strengthened or new* hydrogen-bonding interactions are depicted on mutant/RIT-bound/Ab-bound proteases and *weakened or lost* hydrogen-bonding interactions are depicted on WT-free proteases.

### Hydrophobic contacts and correlation plots

The 80 s loop and the flaps and the flaps of the two monomers themselves form hydrophobic contacts to keep the protease in closed conformation^14,32,33^. In view of this, we assessed the hydrophobic residue contact-pairs present in equilibrated region of the trajectories of WT-free, mutants, RIT-bound and Ab-bound protease. We have also determined the hydrophobic contacts present between the RIT and protease. A hydrophobic-contact is considered to be present, if the distance between any two side-chain carbon atoms is ≤ 0.6 nm. Such criteria are used in literature to define contacts^66,67^. The sampling of these hydrophobic contacts are calculated in percentages using an in-house Tcl script working within VMD TkConsole (http://www-s.ks.uiuc.edu/Research/vmd/vmd-1.9.1/ug/node136.html). Residue pairs having a hydrophobic contact percentage > 10% are obtained. The list of the percentages of the hydrophobic contacts present in mutants, RIT-bound and Ab-bound protease formed between the 80 s loop and the flaps and between the flaps themselves are given in Table S1. Please note that in the WT-free protease there are hardly any hydrophobic contacts involving these regions.

The cross-correlation matrix analysis was performed using GROMACS tools and our own MATLAB script, for Cα atoms of the WT-free, RIT-bound, Ab-bound and mutant proteases. The cross-correlation matrix elements C_ij_ are given by^30,68–71^:

$${{\rm{C}}}_{{\rm{ij}}}={c}_{{\rm{ij}}}/{c}_{{\rm{ii}}}^{1/2}{c}_{{\rm{jj}}}^{1/2}$$

where,

$$\begin{array}{c}{{\rm{c}}}_{{\rm{ij}}}=\langle ({{\bf{r}}}_{i}-\langle {{\bf{r}}}_{i}\rangle )\,({{\bf{r}}}_{j}-\langle {{\bf{r}}}_{j}\rangle )\rangle \\ \,=\langle \Delta {{\bf{r}}}_{{\rm{i}}}.\Delta {{\bf{r}}}_{{\rm{j}}}\rangle \end{array}$$

and

$${{\rm{c}}}_{{\rm{ii}}}=\langle \Delta {{\bf{r}}}_{{\rm{i}}}^{2}\rangle ;\,{{\rm{c}}}_{{\rm{jj}}}=\langle \Delta {{\bf{r}}}_{{\rm{j}}}^{2}\rangle $$

Here, **r**_**i**_ and **r**_**j**_ are Cα atomic coordinates and **∆r**_**i**_ = **r**_i_−〈**r**_i_〉 is a deviation of **r**_**i**_ from average position 〈**r**_i_〉. Similarly **∆r**_**j**_ = **r**_j_−〈**r**_j_〉 is a deviation of **r**_**j**_ from average position 〈**r**_j_〉. The cross-correlation coefficients C_ij_ vary from −1 to +1. The positive values correspond to correlated motions and negative value corresponds to anti-correlated motions. Cross correlation analysis is carried out on Cα coordinates after removing translational and rotational motions with respect to representative conformation from the most populated cluster.

## Supplementary information

Supplementary Information.
