# Supplementary material for: Inhibition of the activity of HIV-1 protease through antibody binding and mutations probed by molecular dynamics simulations
Source: Sci Rep. 2020 Mar 26;10:5501. doi: 10.1038/s41598-020-62423-y (PMC7098958; doi:10.1038/s41598-020-62423-y)
Supplement: Supplementary file 1 — Supplementary Information. [file 41598_2020_62423_MOESM1_ESM.pdf]

Supporting information

for

Inhibition of the activity of HIV-1 protease through antibody binding and mutations probed by molecular dynamics simulations

Apoorva Badaya and Yellamraju U. Sasidhar

Department of Chemistry, Indian Institute of Technology Bombay, Powai, Mumbai 400076, India.

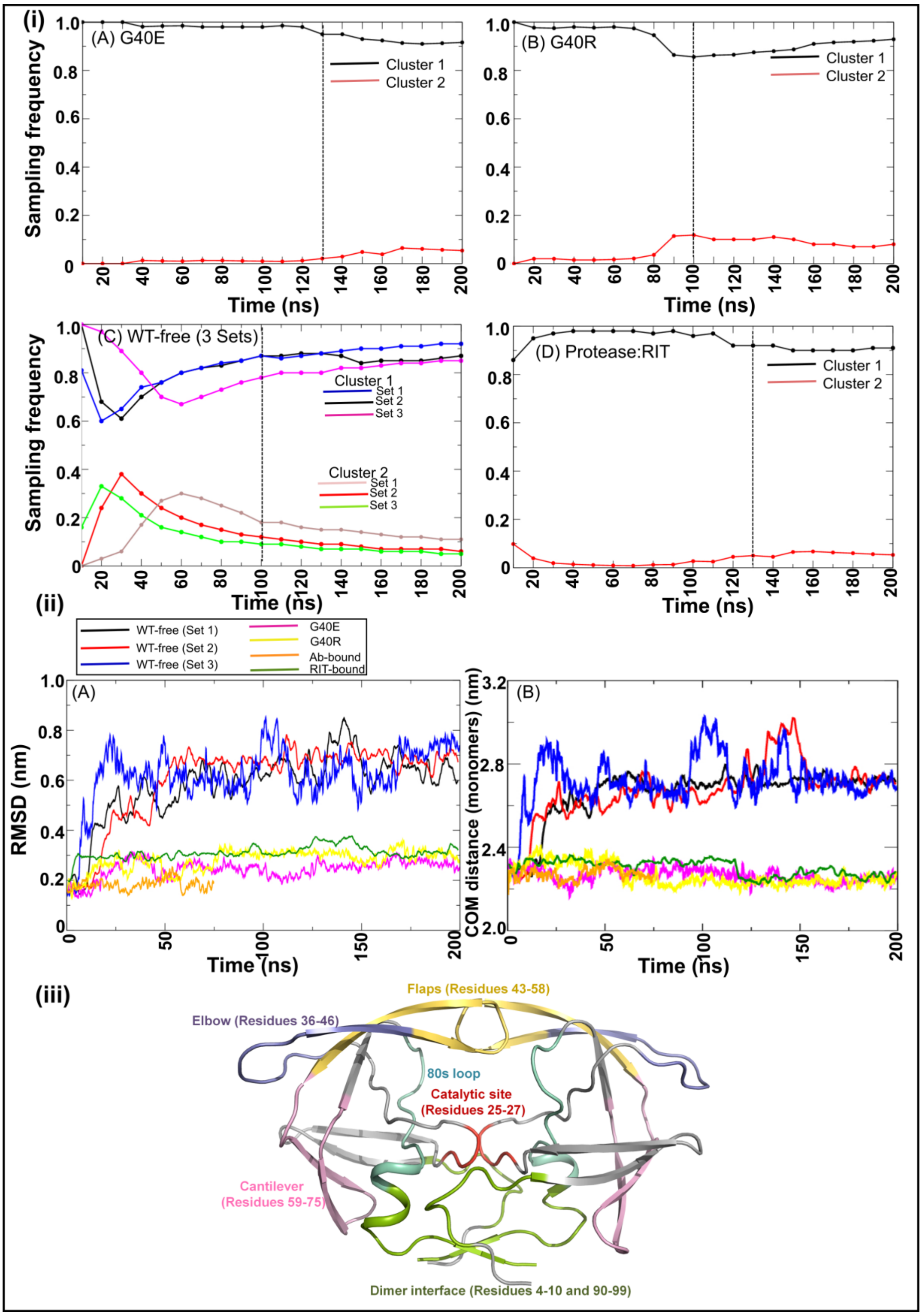

Fig. S1 **(i)** The variation of sampling frequency of the first two most populated clusters (cluster 1 and 2) are determined for the (A) G40E, (B) G40R, (C)WT-free proteases and (D) Protease:RIT complex as a function of the simulation time (see **Methods**). **(ii)** The time variation of **(A)** RMSD with respect to the initial crystal structure conformation and **(B)** centre of mass (COM) distance between the monomers in the Ab-bound, RIT-bound, mutants and 3 sets of the WT-free proteases simulations. **(iii)** The functionally important regions of protease are highlighted on its crystal structure (PDB id: 1HXW).

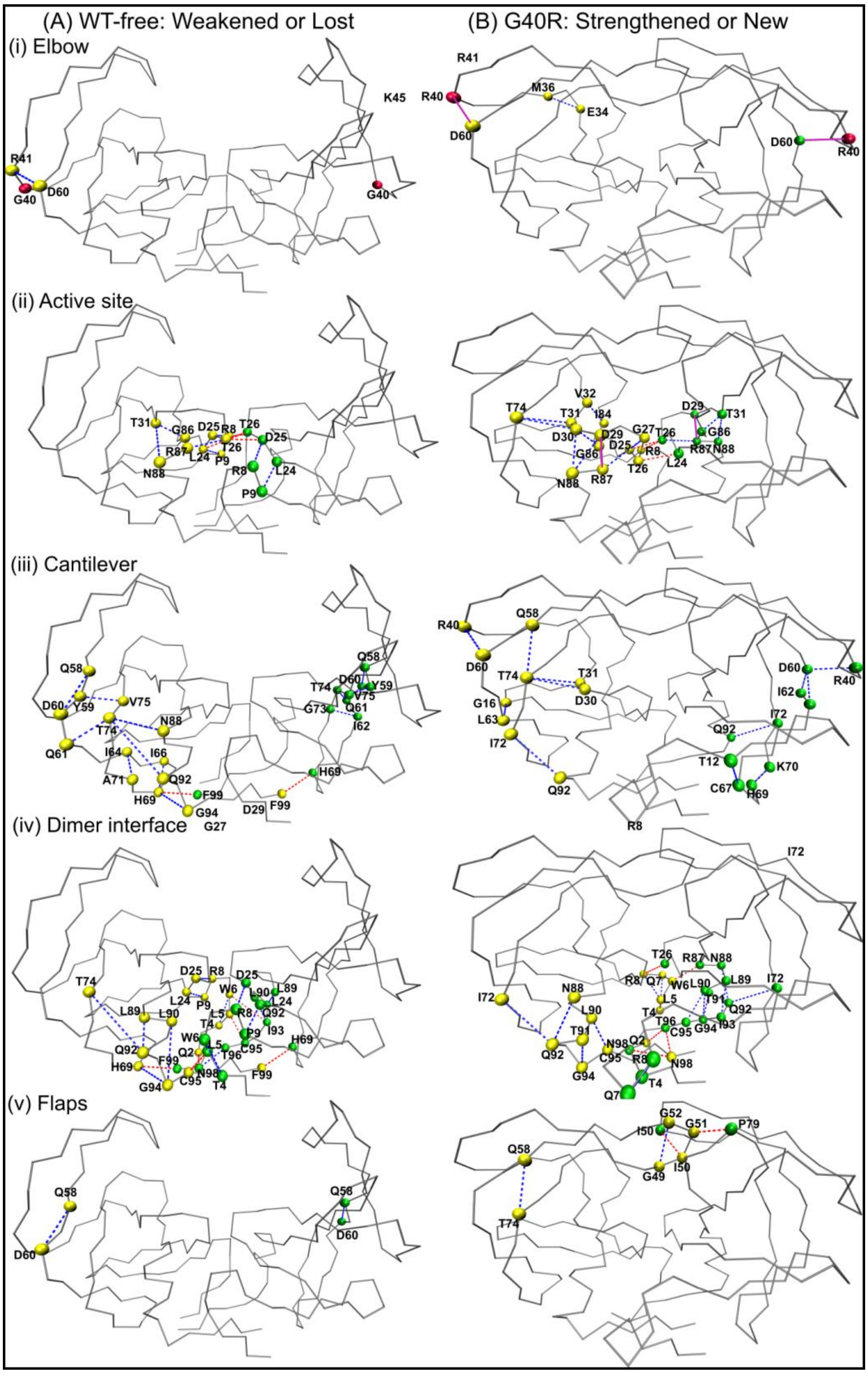

Fig. S2 The representative structure of the (A) WT-free and (B) G40R proteases are shown in trace representation, highlighting and comparing the hydrogen-bonds and salt-bridges that are *weakened or lost* and *strengthened or new* (as explained in **Methods**) for the functionally important regions individually (i) Elbow, (ii), Active site (iii) Cantilever, (iv) Dimer interface and (v) Flaps. The Cα atoms of the residues forming the hydrogen-bond are shown in spheres. The hydrogen-bonds within the monomers are shown as blue dashed line, inter-chain hydrogen-bonds are shown in red dashed-line and the salt-bridges are shown in pink solid line.

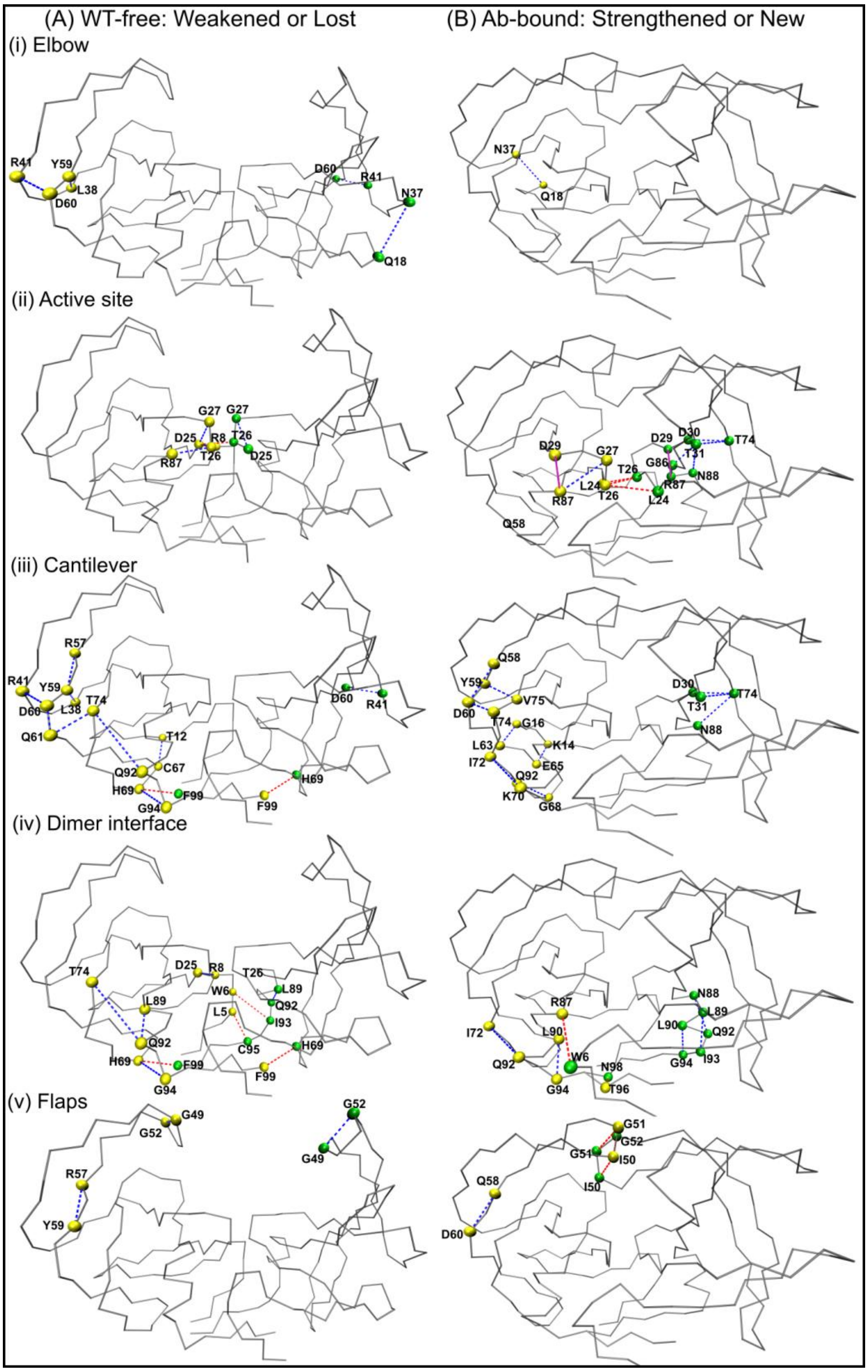

Fig. S3 The representative structure of the (A) WT-free and (B) Ab-bound proteases are shown in trace representation, highlighting and comparing the hydrogen-bonds and salt-bridges that are <u>*weakened or lost*</u> and <u>*strengthened or new*</u> (as explained in **Methods**) for the functionally important regions individually (i) Elbow, (ii), Active site (iii) Cantilever, (iv) Dimer interface and (v) Flaps. The Cα atoms of the residues forming the hydrogen-bond are shown in spheres. The hydrogen-bonds within the monomers are shown as blue dashed line, inter-chain hydrogen-bonds interactions are shown in red dashed-line and the salt-bridges are shown in pink solid line.

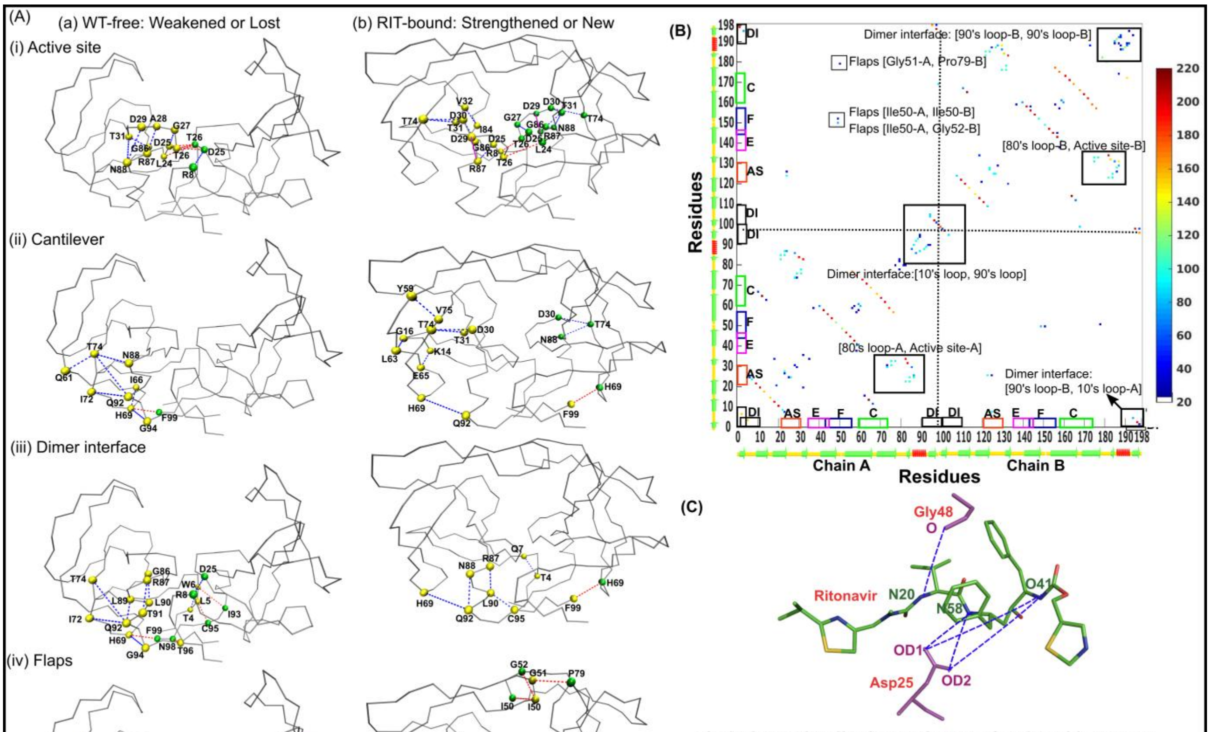

The hydrogen-bonding interactions RIT forming with Protease

S. No	Chain A		
	RIT	Protease	Score
1	N20	G48(A)	47
2	N58	D25 (B)	26
3	O41	D25 (B)	28

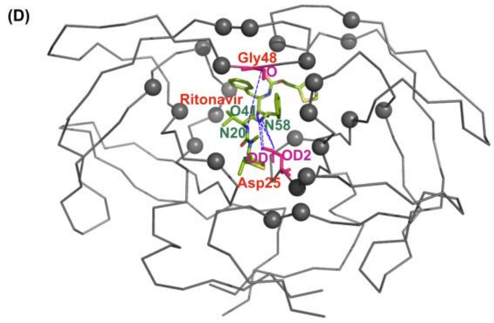

The hydrophobic contacts RIT forming with Protease

S. No	Chain A		Chain B	
	Residue	Percentage	Residue	Percentage
1	A28	99	A28	99
2	V32	98	V32	96
3	I47	99	L23	92
4	I50	99	L24	25
5	I54	69	I47	92
6	F53	42	I50	83
7	I84	99	I54	45
8	P81	93	P81	72
9	V82	32	I84	100
10	P79	44	V82	99
11	L76	37	L76	71
12			P9	34
13			L10	20

Fig. S4 (A) The representative structure of the (a) WT-free and (b) RIT-bound proteases are shown in trace representation, highlighting and comparing the hydrogen-bonds and salt-bridges that are *weakened or lost* and *strengthened or new* (as explained in **Methods**) for the functionally important regions individually (i), Active site (ii) Cantilever, (iii) Dimer interface and (iv) Flaps. In elbow region no *strengthened or new* or *weakened or lost* interactions are observed. The Cα atoms of the residues forming the hydrogen-bond are shown in spheres. The hydrogen-bonds within the monomers are shown as blue dashed line, inter-chain hydrogen-bonds interactions are shown in red dashed-line and the salt-bridges are shown in pink solid line. (B) Hydrogen-bonding map computed from the equilibrated region of the trajectories of RIT-bound proteases. Only those hydrogen-bond pairs are shown, which are having a score more than 20. (C) The residues (shown in magenta) which are forming hydrogen-bonding interactions with RIT (shown in green) are shown in stick representation and the table below lists the atoms of RIT and residues of protease, which are involved in forming the hydrogen-bonding interactions and the corresponding hydrogen-bonding score is given. (D)The representative structure from the Protease:RIT complex simulation is shown, highlighting Cα atoms of the residues forming hydrophobic contacts with the RIT in sphere representation. The residues from protease and RIT involved in forming hydrogen-bonding

interactions are shown in stick representation. The table next to this lists the residues of protease, which are involved in forming hydrophobic contacts with RIT and also the percentage of time the contacts are observed. These residues belongs to active-site, flaps and 80s loop and are highlighted in red, yellow and green color respectively.

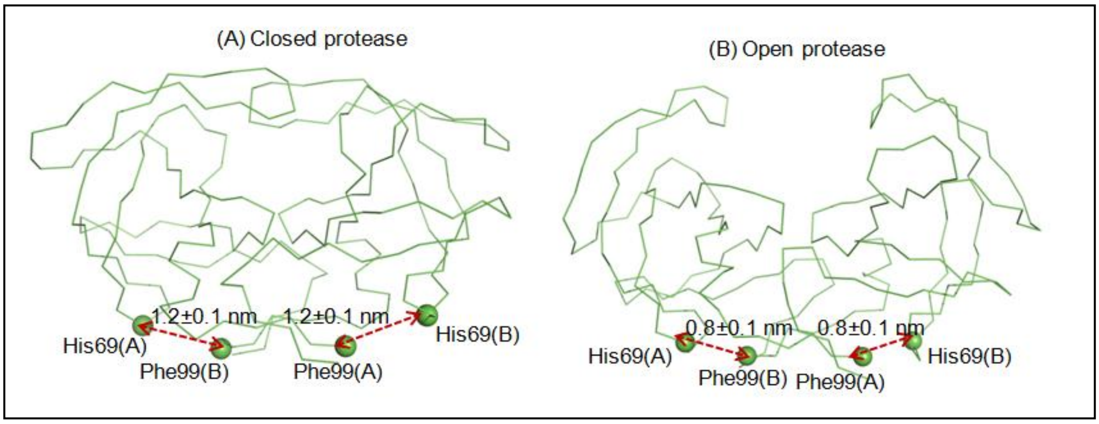

Fig. S5 The distance between the two residues His69 in one chain (at the tip of cantilever) and Phe99 (the terminal residue) in the other chain of protease is assumed to be a reasonable metric to determine the downward motion of the cantilever, upon flap opening. (A) In both the chains of G40E and Ab-bound protease and chain B of G40R and RIT-bound protease this distance is 1.2±0.1 nm and (B) in open WT-free protease this distance is 0.8±0.1 nm.

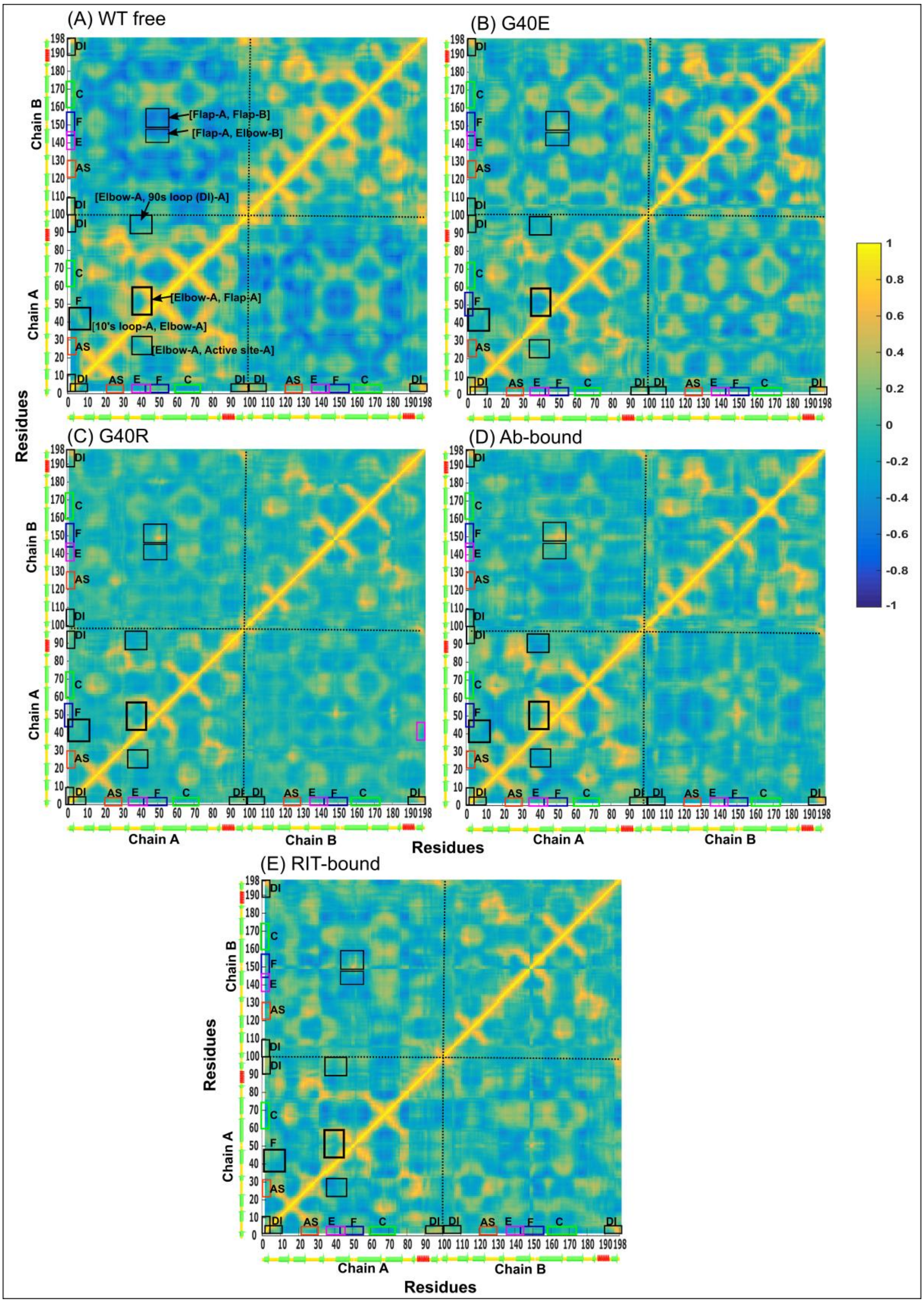

Fig. S6 The cross-correlation plots for the (A) WT-free, (B) G40E, (C) G40R, (D) Ab-bound, (E) RIT-bound proteases obtained for the equilibrated regions of the trajectories. The values of cross-correlation coefficients for positively correlated motions are represented in green to yellow color and similarly the value of cross-correlation coefficients for the anti-correlated motions are represented in light blue to dark blue color. Some of the important highly correlated motions in WT-free protease are highlighted on the map for WT and these are attenuated in mutated, Ab/RIT-bound proteases. Please note that chain labels A and B are also marked. The important regions of protease such as dimer interface (DI), active site (AS), elbow (E), flaps (F) and cantilever (C) are marked on axes of the maps.

Table S1: Percentages of the hydrophobic contacts sampling, present in equilibrated region of the trajectories of mutants, Ab-bound and RIT-bound protease simulations, formed between the 80s loop and flaps (residues 43-58) (highlighted in blue) and between the flaps themselves (highlighted in red). Please note that in the WT-free protease there are hardly any hydrophobic contacts involving these regions.

S. No.	**G40R**		Percentage %		**G40E**		Percentage %
	Chain A	Chain B			Chain A	Chain B	
1	Phe53	Pro81	97		Phe53	Pro81	87
2	Ile50	Ile84	86		Ile50	Ile84	95
3	Pro81	Ile50	18		Pro81	Ile50	82
4	Phe53	Ile50	18		Ile84	Ile50	21
5	Ile54	Ile50	97		Ile54	Ile50	94
6	Ile50	Ile47	89		Ile50	Ile47	78
7	Ile50	Ile54	84		Ile50	Ile54	57
8	Ile50	Ile50	56		Ile50	Ile50	11
9					Ile47	Ile50	16
S. No.	**Ab-bound**		Percentage%		**RIT-bound**		Percentage%
	Chain A	Chain B			Chain A	Chain B	
1	Pro81	Ile50	97		Phe53	Pro81	65
2	Ile 84	Ile 50	95		Pro81	Ile 50	59
3	Pro81	Phe53	42		Ile54	Ile50	88
4	Ile 50	Ile 84	14		Ile50	Ile50	75
5	Ile 50	Pro81	13		Ile50	Ile54	75
6	Val82	Ile 50	13		Ile50	Ile47	53
7	Ile54	Ile 50	96				
8	Ile 50	Ile54	87				
9	Ile 50	Ile 50	51				
10	Ile47	Ile 50	40				
11	Ile 50	Ile47	39				

Table S2: Observed percentages of the salt-bridges present in equilibrated regions of the trajectories of G40R, G40E, Ab-bound and RIT-bound proteases simulations. No salt-bridge sampling is observed in WT-free protease.

Chain	**G40R**		Percentage %	**G40E**		Percentage %
	Donor	Acceptor		Donor	Acceptor	
A	ASP29-OD1	ARG87-NH	60.00	ASP29-OD1	ARG87-NH	57.00
A	ASP29-OD2	ARG87-NH	54.50	ASP29-OD2	ARG87-NH	60.00
B	ASP29-OD1	ARG87-NH	19.00	ASP29-OD1	ARG87-NH	50.00
B	ASP29-OD2	ARG87-NH	38.00	ASP29-OD2	ARG87-NH	55.50
A	ASP60-OD1	ARG40-NH	23.00	GLU40-OE1	ARG41-NH	11.00
A	ASP60-OD2	ARG40-NH	22.50	GLU40-OE2	ARG41-NH	12.50
A	ASP60-OD1	ARG40-NE	8.00	GLU40-OE1	ARG41-NE	10.00
A	ASP60-OD2	ARG40-NE	7.00	GLU40-OE2	ARG41-NE	2.00
B	ASP60-OD1	ARG40-NH	34.00	GLU40-OE1	ARG41-NH	5.50
B	ASP60-OD2	ARG40-NH	34.00	GLU40-OE2	ARG41-NH	5.00
B	ASP60-OD1	ARG40-NE	8.00	GLU40-OE1	ARG41-NE	8.00
B	ASP60-OD2	ARG40-NE	8.00	GLU40-OE2	ARG41-NE	8.00
Chain	**Ab-bound**		Percentage%	**RIT-bound**		Percentage%
A	ASP29-OD1	ARG87-NH	74.00	ASP29-OD1	ARG87-NH	28.00
A	ASP29-OD2	ARG87-NH	75.00	ASP29-OD2	ARG87-NH	28.00
B	ASP29-OD1	ARG87-NH	45.50	ASP29-OD1	ARG87-NH	20.00
B	ASP29-OD2	ARG87-NH	38.50	ASP29-OD2	ARG87-NH	20.00
B	ASP29-OD1	ARG87-NE	17.50			
B	ASP29-OD2	ARG87-NE	12.00			

Table S3: The hydrogen-bonding scores of the *strengthened or new* hydrogen-bonding interactions in G40E with respect to WT-free protease obtained by subtracting the WT hydrogen-bonding map from the G40E hydrogen-bonding map (see **Methods** for definition of *strengthened or new* hydrogen-bonding interactions). The residue pairs forming the hydrogen-bonds in elbows, active-site, cantilever, dimer-interface and flaps are highlighted in gray, red, purple, green and yellow color respectively.

**S. No**	**Chain A**			**Chain B**		
	**Residues**		**Score**	**Residues**		**Score**
1	E40	R41	68.86	E40	R41	65.24
2	E34	M36	32.49	E34	M36	23.20
3	D30	T74	179.90	D30	T74	189.36
4	D30	N88	109.91	D30	N88	109.00
5	D30	G86	82.18	D30	G86	84.09
6	T31	T74	80.52	T31	T74	79.13
7	G27	R87	49.11	R8	L24	51.22
8	V32	I84	43.49	V32	I84	30.20
9	R8	L24	38.33	D29	R87	27.67
10	D29	N88	21.64	D29	N88	21.18
11	I72	Q92	106.86	G27	R87	21.83
12	T4	Q7	81.79	D25	G27	27.70
13	N88	Q92	66.01	I66	G68	20.31
14	L90	C95	42.83	T12	C67	20.21
15	T91	G94	35.75	I72	Q92	34.13
16	G49	G52	24.88	I3	L5	37.23
17	T80	V82	25.86	T4	Q7	57.90
18				T4	W6	23.92
19				N88	Q92	64.70
20				L89	I93	31.66
21				L90	C95	51.86
22				T91	G94	44.44
23				L90	G94	34.93
24				Q58	T74	23.59
25				G49	G52	20.00
			**Interchain**			
**S. No.**	**Chain A**	**Chain B**	**Score**			
1	T26	T26	55.49			
2	N98	N98	56.38			
3	T96	N98	42.66			
4	N98	T96	28.18			
5	T96	Q2	31.59			
6	P79	G51	75.47			
7	G51	P79	75.06			
8	I50	I50	68.34			

Table S4: The hydrogen-bonding scores of the *strengthened or new* hydrogen-bonding interactions in G40R with respect to WT-free protease obtained by subtracting the WT hydrogen-bonding map from the G40R hydrogen-bonding map (see **Methods** for definition of *strengthened or new* hydrogen-bonding interactions). The residue pairs forming the hydrogen-bonds in elbows, active-site, cantilever, dimer-interface and flaps are highlighted in gray, red, purple, green and yellow color respectively.

**S. No**	**Chain A**			**Chain B**		
	**Residues**		**Score**	**Residues**		**Score**
1	E34	M36	31.36	R40	D60	40.75
2	R40	D60	25.98	T26	R87	20.31
3	D25	G27	29.18	T31	G86	56.65
4	G27	R87	75.31	T31	N88	48.18
5	D29	N88	30.85	T12	C67	20.00
6	D30	T74	164.77	D60	I62	23.72
7	D30	N88	97.30	D60	Q61	20.72
8	D30	G86	58.56	H69	K70	20.75
9	D30	T31	38.64	I72	Q92	26.01
10	T31	T74	47.98	T4	R8	91.42
11	V32	I84	36.85	T4	Q7	88.93
12	G16	L63	32.43	N88	Q92	21.63
13	I72	Q92	107.78	L89	I93	34.56
14	T4	Q7	107.89	L90	G94	48.71
15	L5	R8	87.15	L90	C95	28.65
16	N88	Q92	52.52	T91	G94	26.02
17	L90	C95	68.25			
18	T91	G94	53.13			
19	G49	G52	31.91			
20	Q58	T74	25.33			
21	T80	V82	27.57			
22	G86	L89	23.57			
			**Interchain**			
**S. No.**	**Chain A**	**Chain B**	**Score**			
1	R8	T26	71.42			
2	D25	T26	161.91			
3	T26	L24	75.97			
4	Q2	T96	38.17			
5	W6	R87	23.02			
6	R8	T26	71.42			
7	N98	T96	30.38			
8	N98	N98	28.07			
9	I50	I50	75.70			
10	G51	P79	39.84			

Table S5: The hydrogen-bonding scores of the *strengthened or new* hydrogen-bonding interactions in Ab-bound protease with respect to WT-free protease obtained by subtracting the WT hydrogen-bonding map from the Ab-bound hydrogen-bonding map (see **Methods** for definition of *strengthened or new* hydrogen-bonding interactions). The residue pairs forming the hydrogen-bonds in elbows, active-site, cantilever, dimer-interface and flaps are highlighted in gray, red, purple, green and yellow color respectively.

**S. No**	**Chain A**			**Chain B**		
	**Residues**		**Score**	**Residues**		**Score**
1	Q18	N37	64.79	D29	R87	26.10
2	G27	R87	50.52	D30	T74	60.40
3	K14	E65	36.69	T31	N88	115.46
4	G16	L63	76.91	T31	G86	39.98
5	Y59	V75	41.74	T31	T74	20.00
6	D60	T74	21.68	T74	N88	43.95
7	G68	K70	32.94	N88	Q92	20.49
8	I72	Q92	27.62	L89	I93	26.01
9	L90	G94	25.10	L90	G94	77.61
10	Q58	D60	74.87	N88	L89	22.10
11	T80	N83	60.98			
12	N88	L89	20.23			
**Interchain**						
**S. No.**	**Chain A**	**Chain B**	**Score**			
1	L24	T26	42.71			
2	T26	T26	35.72			
3	T26	L24	31.37			
4	R87	W6	51.84			
5	T96	N98	27.10			
6	I50	I50	82.41			
7	I50	G52	26.07			
8	G51	G51	38.43			

Table S6: The hydrogen-bonding scores of the *strengthened or new* hydrogen-bonding interactions in RIT-bound protease with respect to WT-free protease obtained by subtracting the WT hydrogen-bonding map from the RIT-bound protease hydrogen-bonding map (see **Methods** for definition of *strengthened or new* hydrogen-bonding interactions). The residue pairs forming the hydrogen-bonds in active-site, cantilever, dimer-interface and flaps are highlighted in red, purple, green and yellow color respectively.

**S. No**	**Chain A**			**Chain B**		
	**Residues**		**Score**	**Residues**		**Score**
1	D25	R87	93.81	T31	N88	98.47
2	D30	T74	86.16	T31	G86	37.25
3	D30	T31	37.03	G27	D25	30.22
4	V32	I84	35.52	D30	T74	21.07
5	T31	T74	34.78	T74	N88	29.77
6	D30	G86	63.70	N88	L89	28.64
7	H69	Q92	22.28			
8	G16	L63	22.17			
9	K14	E65	21.57			
10	Y59	V75	20.00			
11	T4	Q7	144.01			
12	R87	L90	84.50			
13	N88	Q92	32.79			
14	L90	C95	23.20			
15	G86	N88	61.30			
			**Interchain**			
**S. No.**	**Chain A**	**Chain B**	**Score**			
1	R8	T26	88.63			
2	T26	L24	73.04			
3	F99	H69	24.97			
4	I50	I50	72.86			
5	G52	I50	29.84			
6	G51	P79	27.71			

Table S7: The hydrogen-bonding scores of the *weakened or lost* hydrogen-bonding interactions in G40E with respect to WT-free protease obtained by subtracting the WT hydrogen-bonding map from the G40E hydrogen-bonding map (see **Methods** for definition of *weakened or lost* hydrogen-bonding interactions). The residue pairs forming the hydrogen-bonds in elbows, active-site, cantilever, dimer-interface and flaps are highlighted in gray, red, purple, green and yellow color respectively.

**S. No**	**Chain A**			**Chain B**		
	**Residues**		**Score**	**Residues**		**Score**
1	R41	D60	50.34	R41	D60	37.62
2	T31	N88	135.40	T31	N88	39.11
3	T31	G86	93.13	T31	G86	30.75
4	T26	R87	48.79	T26	R87	35.27
5	P9	L24	73.86	P9	L24	70.34
6	R8	D25	26.37	R8	D25	36.99
7	T74	Q92	55.63	I66	H69	40.10
8	T74	N88	44.04	I64	A71	30.10
9	H69	G94	26.54	I62	G73	23.62
10	I66	H69	28.05	Q61	T74	21.42
11	I64	A71	29.17	G16	L63	23.03
12	Q61	T74	44.56	T96	N98	23.77
13	T96	N98	23.11	L90	I93	20.57
14	L89	Q92	19.79	L89	Q92	37.95
15	Q58	D60	38.61	Q58	D60	25.41
**Interchain**						
**S. No.**	**Chain A**	**Chain B**	**Score**			
1	L24	T26	39.39			
2	F99	H69	58.75			
3	H69	F99	22.58			
4	N98	Q2	28.73			
5	C95	L5	91.79			
6	W6	I93	21.1			
7	L5	C95	100.37			
8	I3	L97	29.63			
9	Q2	N98	34.96			

Table S8: The hydrogen-bonding scores of the *__weakened or lost__* hydrogen-bonding interactions in G40R with respect to WT-free protease obtained by subtracting the WT hydrogen-bonding map from the G40R hydrogen-bonding map (see **Methods** for definition of *weakened or lost* hydrogen-bonding interactions). The residue pairs forming the hydrogen-bonds in elbows, active-site, cantilever, dimer-interface and flaps are highlighted in gray, red, purple, green and yellow color respectively.

**S. No**	**Chain A**			**Chain B**		
	**Residues**		**Score**	**Residues**		**Score**
1	R41	D60	20.24	R8	D25	55.08
2	R8	D25	32.55	P9	L24	95.19
3	P9	L24	27.23	I62	G73	80.44
4	T26	R87	29.45	Q61	T74	24.59
5	T31	N88	135.40	Y59	V75	53.28
6	T31	G86	87.71	T4	W6	21.79
7	T74	Q92	55.63	L89	Q92	36.38
8	T74	N88	44.04	T96	N98	24.17
9	H69	G94	26.54	L90	I93	22.26
10	I66	H69	20.20	Q58	D60	29.38
11	I64	A71	20.24			
12	Q61	T74	43.76			
13	Y59	V75	21.87			
14	T4	W6	23.80			
15	L89	Q92	20.00			
16	L90	G94	20.99			
17	Q58	D60	41.98			
**Interchain**						
**S. No.**	**Chain A**	**Chain B**	**Score**			
1	L24	T26	39.39			
2	T26	T26	136.26			
3	T26	D25	51.42			
4	F99	H69	29.01			
5	H69	F99	22.58			
6	Q2	N98	29.40			
7	L5	C95	70.40			
8	W6	I93	21.1			

Table S9: The hydrogen-bonding scores of the *weakened or lost* hydrogen-bonding interactions in Ab-bound protease with respect to WT-free protease obtained by subtracting the WT hydrogen-bonding map from the Ab-bound hydrogen-bonding map (see **Methods** for definition of *weakened or lost* hydrogen-bonding interactions). The residue pairs forming the hydrogen-bonds in elbows, active-site, cantilever, dimer-interface and flaps are highlighted in gray, red, purple, green and yellow color respectively.

**S. No**	**Chain A**			**Chain B**		
	**Residues**		**Score**	**Residues**		**Score**
1	R41	D60	66.32	R41	D60	32.78
2	L38	Y59	31.19	Q18	N37	27.09
3	R8	D25	50.44	D25	G27	25.66
4	D25	G27	46.87	L89	Q92	35.34
5	T26	R87	29.75	G49	G52	42.04
6	T74	Q92	55.63			
7	D60	Q61	47.89			
8	T12	C67	44.70			
9	H69	G94	26.54			
10	Q61	T74	26.37			
11	L89	Q92	20.72			
12	R57	Y59	34.24			
13	G49	G52	21.58			
**Interchain**						
**S. No.**	**Chain A**	**Chain B**	**Score**			
1	D25	T26	21.37			
2	F99	H69	41.36			
3	H69	F99	22.58			
4	L5	C95	45.36			
5	W6	I93	21.1			

Table S10: The hydrogen-bonding scores of the *weakened or lost* hydrogen-bonding interactions in RIT-bound protease with respect to WT-free protease obtained by subtracting the WT hydrogen-bonding map from the RIT-bound hydrogen-bonding map (see **Methods** for definition of *weakened or lost* hydrogen-bonding interactions). The residue pairs forming the hydrogen-bonds in active-site, cantilever, dimer-interface and flaps are highlighted in red, purple, green and yellow color respectively.

**S. No**	**Chain A**			**Chain B**		
	**Residues**		**Score**	**Residues**		**Score**
1	T31	N88	135.12	R8	D25	55.08
2	A28	R87	94.31	R57	V77	20.97
3	T31	G86	90.69			
4	D29	N88	80.61			
5	D25	G27	25.55			
6	I72	Q92	74.59			
7	T74	Q92	55.63			
8	T74	N88	44.04			
9	Q61	T74	41.73			
10	H69	G94	26.54			
11	I66	H69	19.98			
12	R87	T91	113.67			
13	G86	L90	78.19			
14	T4	W6	21.26			
15	L89	Q92	20.00			
16	R57	V77	32.34			
17	T80	V82	45.46			
18	G86	L89	24.52			
**Interchain**						
**S. No.**	**Chain A**	**Chain B**	**Score**			
1	T26	T26	106.88			
2	T26	D25	51.41			
3	L24	T26	39.49			
4	D25	T26	20.00			
5	H69	F99	22.58			
6	L5	C95	28.27			
7	T96	N98	23.90			
8	W6	I93	20.49			
